# Impact of crowded environments on binding between protein and single-stranded DNA

**DOI:** 10.1038/s41598-021-97219-1

**Published:** 2021-09-03

**Authors:** Birgit Köhn, Patricia Schwarz, Pernilla Wittung-Stafshede, Michael Kovermann

**Affiliations:** 1 Department of Chemistry, University of Konstanz, Universitätsstrasse 10, 78457 Konstanz, Germany; 2 Konstanz Research School Chemical Biology KoRS-CB, University of Konstanz, Universitätsstrasse 10, 78457 Konstanz, Germany; 3 Department of Biology and Biological Engineering, Chalmers University of Technology, 41296 Gothenburg, Sweden

**Keywords:** Biopolymers in vivo, Biophysical chemistry

## Abstract

The concept of Molecular Crowding depicts the high density of diverse molecules present in the cellular interior. Here, we determine the impact of low molecular weight and larger molecules on binding capacity of single-stranded DNA (ssDNA) to the cold shock protein B (CspB). Whereas structural features of ssDNA-bound CspB are fully conserved in crowded environments as probed by high-resolution NMR spectroscopy, intrinsic fluorescence quenching experiments reveal subtle changes in equilibrium affinity. Kinetic stopped-flow data showed that DNA-to-protein association is significantly retarded independent of choice of the molecule that is added to the solution, but dissociation depends in a nontrivial way on its size and chemical characteristics. Thus, for this DNA–protein interaction, excluded volume effect does not play the dominant role but instead observed effects are dictated by the chemical properties of the crowder. We propose that surrounding molecules are capable of specific modification of the protein’s hydration shell via soft interactions that, in turn, tune protein–ligand binding dynamics and affinity.

## Introduction

Specific interactions of proteins with DNA take place in cells in the presence of up to 400 g/L of macromolecules^1,2^, and the environments are additionally confined by cellular and compartmental membranes. Recently published studies have shown that cellular compartments can be further segregated into membraneless bodies of specific composition with even higher local concentrations of different biopolymers^3–5^. Specific protein-to-DNA interactions are tightly regulated by the affinities (which is a combination of association and dissociation rate constants) of the respective binding partners which strongly depend on effective local concentrations^6^ as well as viscosity dependent diffusion^7^ of the binding partners. Further alterations of affinity may occur due to more subtle effects such as sticking of the binding partners to any encountered obstacle in solution caused by unspecific electrostatic, hydrophobic or van der Waals interactions, and a sterically induced “excluded volume” effect. The term “excluded volume” was originally coined by Werner Kuhn to describe the volume around the center of an object which cannot be penetrated by the center of another object^8^ and further applied by Paul Flory to the self-exclusion of segments in a polymer chain^9^. Minton, who coined the expression “macromolecular crowding” in 1981^10^, refers with this to the effect that volume exclusion has on the energetics and transport properties of macromolecules in a highly volume-occupied medium^11^, where the reduction of the available volume will favor compact over expanded conformations as well as associated over dissociated states. In recent years of macromolecular crowding research this classical crowding concept focusing merely on “hard interaction” (steric, entropic effects) has been acknowledged to be modified by so-called “soft interactions” (enthalpic effects)^12^. In order to estimate the role of chemical properties of the macromolecular crowders in context of the overall crowding effect, it is required to include a focus on the effects caused by the monomeric subunits of the macromolecules, giving rise to the concept of “low molecular weight crowders”^13^. This aspect directly overlaps with the well-established concepts of solvency and osmolyte effects in the way of treating cosolutes^14^. On the one hand, excluded volume has been considered in model calculations to influence the entropic component of protein folding and association reactions in solution^15–19^. Excluded volume gives rise to depletion interactions, which occur when the excluded volumes of macromolecular particles overlap, resulting in macromolecules repelling other molecules from that space, generating—via osmotic pressure changes—an attractive force between the particles that initially overlap in their excluded volumes. This will cause association as soon as the attractive interaction is strong enough to overcome the loss in conformational entropy^20^. Note that colloidal particles and polymer chains may generate differences in the overlap volume due to their inherent differences in three-dimensional conformational space. On the other hand, effects such as unspecific binding, hydrophobic or polar contact interactions between macromolecules and cosolutes have been considered to account for enthalpic changes in thermodynamic parameters^21,22^. Here the term “hydrophobic interactions” refers to the attractive interaction taking place when hydrophobic patches of macromolecules or hydrophobic residues get in contact with each other. This interaction can be further modified by solvent properties, for example hydrophobic solvent moieties can reduce the hydrophobic interaction between the macromolecules by offering alternative hydrophobic interaction sites, whereas highly polar solvents can enhance the hydrophobic interaction between macromolecules. As one striking example, the influence of polyethylene glycol on hydrophobic base-pair stacking in DNA was observed recently^23^. Besides that, effects of cellular confinement on kinetic interactions have been considered in different models dealing with fractal environments^24^ and depletion interactions (representing an attractive force between particles that is based on the exclusion of solutes from the proximity of these two particles, also noted above) depending on the concentration and size of particles (inert molecules) constituting the solution under study^20,25^.

In contrast to the complexity of the cellular environment, affinities of DNA–binding proteins are usually determined in vitro at experimental conditions comprising a dilute solution of a buffering agent and purified interaction partners stripped of their cellular surrounding. Parameters determined under such a diluted setting cannot hold for in vivo interactions and have to be corrected in order to draw realistic conclusions about the kind and strength of interactions that take place in vivo. On the one hand, the study of affinities at standard laboratory conditions is reasonably established due to the limited resolution and timely restrictions of measurements in vivo. On the other hand, due to the limited resolution and timely restrictions of measurements in vivo, the acquisition of highly resolved experimental data for individual protein species, which are present at defined crowded conditions in vitro, represents one rational way of considering the intracellular complexity. Up to date though, a consent regarding a valid model of how to mimic the cellular environment in the test tube is missing.

In order to transfer binding affinities for ligand-to-protein interactions as well as conformational stabilities and kinetic data derived from high resolution in vitro measurements to the diverse, highly crowded cellular environment, a bottom-up approach is appropriate^26,27^. This strategy accounts for the multiple and diverse intracellular contributing factors by assessing them one by one. Thus, starting from dilute experimental conditions, the controlled addition of inert molecules (often referred to as ‘crowding agents’) possessing individual physical and chemical properties to the test tube enables us to mimic the density of the intracellular environment^28,29^ and, at the same time, suppress the natural sample degradation and additional simultaneous interactions with multiple binding partners that could take place in vivo. Advantageously, this bottom-up approach focuses on one (bio)physical parameter at a time, and allows us to consider steric interactions apart from charged or strongly polar interactions by the choice of added molecule and enables the investigation of a selected interaction between two binding partners with highly-resolved biophysical methods. Here, we set out to probe the effect of a crowded environment, using large as well as small molecules, on a selected DNA-to-protein interaction.

To implement crowded environments, we have used concentrations of 100–300 g/L of the macromolecules polyethylene glycol of 1 or 8 kDa (PEG1, PEG8), and dextran of 20 kDa molecular mass (Dex20) and their monomeric counterparts ethylene glycol (EG) and glucose. As the DNA–protein pair, we have examined the interaction between the nucleic acid binding protein CspB (Cold shock protein B) from *Bacillus subtilis* (*Bs*CspB) and ssDNA (single stranded deoxyribonucleic acid) of various length. *Bs*CspB is a well-investigated protein as the highly resolved three-dimensional structure of its ligand-free state has been known for long^30,31^. It has been shown that *Bs*CspB binds preferentially to 6–7 nucleotide (nt) long stretches of thymine (T)-based ssDNA^32^. Zeeb et al. characterized the binding interface between *Bs*CspB and ssDNA by solution state NMR spectroscopy^33^ and the solution structure of *Bs*CspB in complex with heptathymidine (TTTTTTT, dT7) has been solved^34^. Further, a crystal structure of *Bs*CspB bound to dT6^35,36^ and structures of *Bs*CspB bound to several RNA molecules have been reported^37^. Moreover, the impact of a crowded environment on the thermodynamic stability of *Bs*CspB was recently probed on the atomic-level by using a convergent experimental setup comprising different biophysical techniques including fluorescence and high-resolution NMR spectroscopy^26,27^. The presence of crowded environments increases the thermodynamic stability of this protein and thus it is of high interest to explore the effects of such crowded environments also on the interaction between *Bs*CspB and oligonucleotides.

In this study we have combined high-resolution NMR and fluorescence spectroscopy, in equilibrium and time-resolved, to probe the site, strength, and dynamics of this protein–DNA interaction in a set of differently crowded environments. We found by intertwining high-resolution NMR with fluorescence spectroscopy that the presence of a crowded environment significantly retards the association kinetics between single stranded DNA and *Bs*CspB, independent of the inherent characteristics and size of the added molecule. Contrary, the change in ligand dissociation comparing dilute conditions with crowded environments depends precisely on the chemical features of the molecule that is supplemented to the solution under study. The nontrivial results obtained in this study highlight that excluded volume effects do not dominate but instead solvation effects, provided by both low molecular weight and larger crowding molecules in a chemistry-dependent way, play a large role. Due to the fundamental importance of protein–DNA interactions for cell functions, and the widespread variations of existing pairs of proteins and oligonucleotides found in biology^38–40^, our study on one selected DNA–protein pair has broad implications.

## Results

### Crowded environments do not modify *Bs*CspB structure when bound to ssDNA

**Figure 1:**
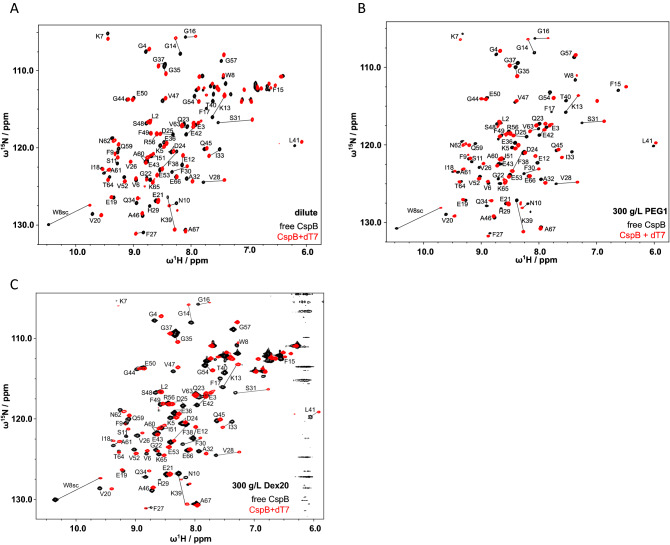
Probing the interaction between dT7 and *Bs*CspB in absence and presence of a crowded environment by high-resolution NMR spectroscopy. Two-dimensional heteronuclear ^1^H–^15^N HSQC spectra comparing free (colored in black) and dT7-bound *Bs*CspB (colored in red) under dilute conditions (**A**) and in presence of 300 g/L PEG1 (**B**) or 300 g/L Dex20 (**C**). The assignment of backbone signals of *Bs*CspB is indicated by using the one letter code for amino acids followed by the position in the primary sequence. A twofold molar excess of dT7 regarding *Bs*CspB has been used to populate the respective ligand-bound state of *Bs*CspB. Protein concentrations varied between 150 µM (free *Bs*CspB) and 144 µM (*Bs*CspB + dT7). NMR spectra shown in (**A**) and (**B**) have been acquired at *T* = 298 K, *B*_0_ = 18.8 T, spectra shown in (**C**) have been acquired at *T* = 294.4 K, *B*_0_ = 14.1 T, respectively. Analysis of chemical shift perturbations (CSPs) is presented as associated data in Fig. S1C–J.

First, we applied high-resolution NMR spectroscopy to obtain residue-specific insights into the structural and dynamical impact of crowded environments upon stepwise addition of dT7 to ^13^C and ^15^N isotopically labelled *Bs*CspB. Changes of chemical shifts, signal heights and line widths of resonance signals upon complex formation were analyzed based on the acquisition of heteronuclear two-dimensional ^1^H–^15^N and ^1^H–^13^C HSQC as well as one-dimensional ^31^P spectra. Qualitatively, the spectra acquired at start—in the absence of dT7—and at end of the titration experiment—at a two-fold molar excess of dT7 over *Bs*CspB—are comparable in the absence or presence of 300 g/L PEG1 or Dex20 (Fig. 1A–C). Only minor perturbations of chemical shifts occur, indicating no significant changes in the *Bs*CspB-dT7 complex comparing a crowded environment to dilute conditions. Importantly, this result holds for analyses of the protein backbone (Figs. 1A–C, S1A–J), cross-peaks of side chain resonances (Fig. S1A,B), and the phosphate backbone resonances in dT7 (Fig. S1K,L). Comparing free with dT7-bound protein states at dilute conditions, perturbations of ^1^H and ^15^N chemical shifts (CSPs) of resonance signals comprising *Bs*CspB are exceeding the mean plus one standard deviation predominantly for Gly14, Val28, Ser31, Phe38, Lys39, and Thr40 (Fig. S1C,H). Those identified residues are in full agreement with known sites of perturbations for the hexathymidine (dT6)-*Bs*CspB complex under dilute conditions^34^. Perturbations of chemical shifts report inherently either on a direct interaction of residues that contribute to the binding interface of the protein under study with the ligand, or on a remote conformational change providing a cooperative or allosteric effect^33^. The former is the case for Phe38 since this residue is involved in base stacking with nucleobases of the ligand^36^ whereas the latter can be seen by moderate perturbations of chemical shifts of residues Glu50–Gly54 that are located distant from the binding interface^33^. It has also been shown by site-directed mutagenesis that the mobility of residue Arg56 is essential for high affinity ligand binding^34^. In 300 g/L PEG1 or 300 g/L Dex20, chemical shift perturbations upon addition of a twofold excess of dT7 report on a pattern highly similar to the one that is observed under dilute conditions (Fig. S1D,I). Correlation analyses of chemical shift perturbations (CSPs) under dilute conditions and in crowded environments (presence of PEG1 or Dex20 molecules) reveals an almost linear dependence (Fig. S1F). Thus, CSP analysis demonstrates no difference in the protein–DNA complex structure when dilute conditions are compared with crowded environments.

The above conclusion is corroborated by analyses of individual signal heights and line widths of the two-dimensional ^1^H–^15^N HSQC NMR spectra. When comparing the ratio of signal heights found for start- and endpoints of the titration experiments conducted under dilute conditions as well as in various crowded environments, a qualitatively similar overall pattern on a residue-by-residue basis can indeed be observed (Fig. S2A–E). The mean ratio of intensities found for dilute conditions, *I*_end_/*I*_start_ = 1.6 ± 1.7 (Fig. S2A), slightly decreases when the concentration of crowding molecules increases to 100 g/L PEG8 (*I*_end_/*I*_start_ = 1.5 ± 0.7, Fig. S2B), 200 g/L PEG8 (*I*_end_/*I*_start_ = 1.4 ± 0.8, Fig. S2C), 300 g/L PEG1 (*I*_end_/*I*_start_ = 1.1 ± 0.8, Fig. S2D), and 300 g/L Dex20 (*I*_end_/*I*_start_ = 1.2 ± 0.4, Fig. S2E). Most residues in *Bs*CspB show progression patterns that can be categorized into the slow-to-intermediate exchange regime on the NMR time scale^41^. For the majority of cross-peaks, the exchange regime is not altered in presence of crowding agents as compared to dilute conditions (Fig. S4). The only cross-peaks showing significant differences in signal heights over the course of the NMR titration series when comparing dilute with crowded conditions are Phe9, Val26, Glu53, Arg56, and Gly57, respectively (Fig. S3D–I). Note that none of these residues showed pronounced changes in chemical shifts upon addition of dT7 (Fig. S1C–E).

The line width of NMR resonance signals enables the evaluation of the binding between *Bs*CspB and dT7 in terms of dynamic processes^33^. The analysis of the line width in the proton dimension of 17 residues comprising the binding interface between dT7 and *Bs*CspB yields a mean of 36 ± 6 Hz in absence of dT7 applying dilute conditions (Fig. S5A). A twofold stoichiometric excess of dT7 over *Bs*CspB decreases the mean in line width by about ten percent to 32 ± 2 Hz indicating a highly dynamic binding process (Fig. S5A). In presence of 300 g/L PEG1, the mean in line width is specified with 38 ± 2 Hz when analyzing the same 17 residues comprising *Bs*CspB in absence of dT7 (Fig. S5B). Interestingly, a twofold excess of dT7 over *Bs*CspB does not modify the mean line width in presence of 300 g/L PEG1, as it again amounts to 38 ± 4 Hz (Fig. S5B). The analysis of line widths in the proton dimension of the same 17 residues performed in presence of 300 g/L Dex20 leads to 42 ± 4 Hz in absence of dT7 and 40 ± 4 Hz in presence of a twofold stoichiometric excess of dT7, respectively (Fig. S5C). This invariance or only slight decrease in line width of residues comprising the binding interface between *Bs*CspB and dT7 hints at alterations in the dynamics of binding comparing dilute with crowded conditions. Note that the significant increase in solvent viscosity as induced by the addition of 300 g/L PEG1 or 300 g/L Dex20 does not per se increase the line width of residues in *Bs*CspB as no major differences in line widths can be observed when ligand-free states are compared (Fig. S5A–C).

Summing up, the analysis of NMR titration data in terms of chemical shifts, signal heights and line widths of cross-peaks reveals modest changes in ligand binding upon interaction of dT7 with *Bs*CspB when dilute conditions and crowded environments are compared. The dT7-bound state of *Bs*CspB is retained in crowded environments but the data reveal some changes in ligand binding dynamics. As the affinity, *K*_D_, between dT7 and *Bs*CspB is expected to be in the low nanomolar range ^34^, and considering stoichiometric binding at conditions used in NMR titration experiments, this approach cannot unravel changes in *K*_D_ values that may be induced by a crowded environment. Consequently, fluorescence spectroscopy was subsequently employed to monitor protein-to-ssDNA association and dissociation kinetics at much lower concentrations of DNA and protein.

### Particular crowded environments decrease affinity of ssDNA to *Bs*CspB

**Figure 2:**
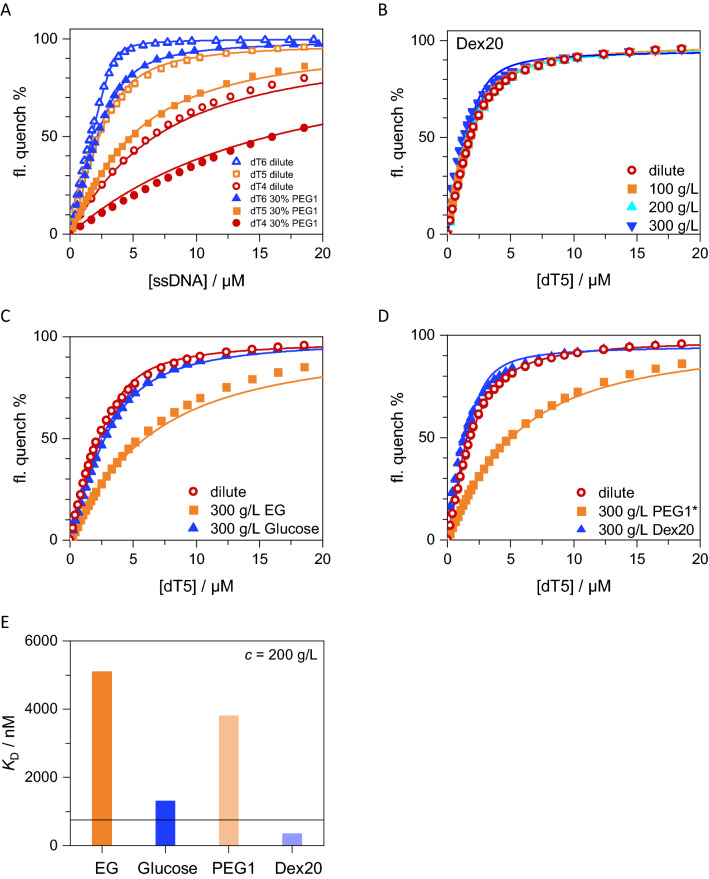
Probing the interaction between different oligothymidines and *Bs*CspB in absence and presence of a crowded environment by applying fluorescence spectroscopy in equilibrium. (**A**) The quench of intrinsic fluorescence of *Bs*CspB has been determined for adding dT4 (colored in red, circles), dT5 (colored in orange, rectangles), and dT6 (colored in blue, triangles) under dilute (open symbols) and 300 g/L PEG1 conditions (closed symbols). (**B**) Intrinsic fluorescence quenching has been determined for adding dT5 in absence (colored in red, circles) and presence of 100 g/L Dex20 (colored in orange, rectangles), 200 g/L Dex20 (colored in cyan, triangles with tip to top), and 300 g/L Dex20 (colored in blue, triangles with tip to bottom). (**C**) Intrinsic fluorescence quenching has been determined for adding dT5 in absence (colored in red, circles) and presence of 300 g/L EG (colored in orange, rectangles) and 300 g/L glucose (colored in blue, triangles). (**D**) Intrinsic fluorescence quenching has been determined for adding dT5 in absence (colored in red, circles) and presence of 300 g/L PEG1 (colored in orange, rectangles) and 300 g/L Dex20 (colored in blue, triangles). All experiments have been performed at *T* = 298 K using *c*^*Bs*CspB^ = 4 µM, except in (**D**) where *c*^*Bs*CspB^ = 2.8 µM was used for *c*^PEG1^ = 300 g/L. Equation (3) has been applied to perform numerical analysis of experimental data and the corresponding fitting functions are shown as continuous lines. (**E**) Binding affinity of dT5 to *Bs*CspB in presence of *c* = 200 g/L of EG, glucose, PEG1, or Dex20. The horizontal line indicates binding affinity under dilute conditions. Associated data reporting on the binding affinity between oligothymidines and *Bs*CspB are presented in Table 1 and Fig. S6A–E.

We applied fluorescence spectroscopy to investigate effects of crowded environments on the affinity of ssDNA to *Bs*CspB. Titration experiments using steady state fluorescence spectroscopy showed that a change in affinity of oligothymidines to *Bs*CspB is dependent on the type and concentration of the crowding agent (Fig. 2A–D, Table 1). The presence of e.g. 300 g/L PEG1 leads to a significant decline in the affinity of oligothymidines regarding *Bs*CspB as seen for tetra-, penta-, hexa-, and heptathymidines compared to dilute conditions (Figs. 2A, S6A–C, Table 1). The magnitude of change in affinity shows different extents for oligothymidines differing in length (Table 1). A modest decline in ligand binding affinity by a factor of three is seen for dT4 (Table 1) but a decrease by a factor of about 30 is seen for dT7, when comparing presence of 300 g/L PEG1 to dilute conditions (Table 1). Note that the gradual increase in *K*_D_ values determined for oligothymidines of decreasing length observed under dilute conditions is conserved in the crowded environment constituted by PEG1 (Table 1). However, the dependence of *K*_D_ values on the length of the ligand interacting with *Bs*CspB observed under dilute conditions^34,37^ is exaggerated in presence of crowded environments (Table 1).

**Table 1:** Numerical values reporting on the binding affinity, *K*_D_, of different oligonucleotides to *Bs*CspB comparing dilute conditions with crowded environments determined by fluorescence spectroscopy in equilibrium.

Ligand	Condition	*K*_D_/nM	*K*_D_^crowd^/*K*_D_^dilute^
dT4	Dilute	6100 ± 300	
dT5	Dilute	750 ± 50	
dT5^b^	Dilute	780 ± 10	
dT6	Dilute	80 ± 10	
dT6^a^	Dilute	30 ± 10	
dT7	Dilute	15 ± 5	
dT7^a^	Dilute	7 ± 3	
dT4	300 g/L PEG1	17000 ± 1100	3
dT5	300 g/L PEG1	3800 ± 100	5
dT6	300 g/L PEG1	580 ± 10	7
dT6^a^	300 g/L PEG1	415 ± 5	14
dT7	300 g/L PEG1	570 ± 30	41
dT7^a^	300 g/L PEG1	170 ± 5	24
dT5^b^	100 g/L Dex20	710 ± 20	0.9
dT5^b^	200 g/L Dex20	680 ± 20	0.9
dT5^b^	300 g/L Dex20	350 ± 50	0.5
dT5	300 g/L EG	5100 ± 400	7
dT5	300 g/L glucose	1310 ± 20	1.7
dT7^b^	100 g/L PEG1	10 ± 10	1
dT7	100 g/L PEG8	14 ± 7	1
dT7	200 g/L PEG1	75 ± 15	5
dT7	200 g/L PEG8	90 ± 20	8
dT7^b^	300 g/L Dex20	6 ± 3	0.5
dT7^a^	300 g/L Dex20	5 ± 3	0.5

The concentration of *Bs*CspB was set to 0.5 µM (indicated with ^a^), 2.8 µM (indicated with ^b^) or 4 µM in fluorescence experiments which have been performed at *T* = 298 K. *K*_D_^crowd^: applying a crowded environment. *K*_D_^dilute^: applying dilute conditions. Associated data are shown in Figs. 2A–D and S6A–E.

For separation of the size dependent macromolecular crowding effect from effects due to the chemical properties of the respective crowder, we also tested the effects of 300 g/L low molecular weight EG—a subunit of the polymeric crowding agent PEG—on binding between oligothymidines and *Bs*CspB. The *K*_D_ value observed for the binding of dT5 to *Bs*CspB in presence of 300 g/L PEG1 is comparable to the value obtained in presence of 300 g/L EG (Fig. 2E, Table 1). This suggests that the effects on the binding affinity found for adding PEG1 or PEG8 to the test tube are not primarily excluded volume effects. Therefore, we tested another macromolecular crowding agent possessing different chemical properties to PEG, dextran, and its low molecular weight counterpart, glucose. Using solutions comprising 100 g/L Dex20, 200 g/L Dex20, and 300 g/L glucose to probe binding of dT5 to *Bs*CspB, we found that neither Dex20 nor glucose significantly modifies the binding affinity as compared to dilute conditions (Fig. 2E, Table 1). The *K*_D_ values were determined to *K*_D_^300^ ^g/L,glucose^ = 1.31 ± 0.02 μM and *K*_D_^200^ ^g/L,Dex20^ = 0.68 ± 0.02 μM, respectively, and can be compared to *K*_D_^dilute^ = 0.8 ± 0.1 μM. Thus, the *K*_D_ value for the interaction between dT5 and *Bs*CspB remains almost constant when using glucose or Dex20 molecules contrarily to the effects observed for the presence of EG, PEG1, or PEG8. The same results were found when using dT7 instead of dT5 as ligand (Fig. S6E, Table 1).

Overall, fluorescence equilibrium experiments revealed that the affinity of an oligothymidine to *Bs*CspB decreases when comparing dilute conditions with an environment that is constituted by EG, PEG1, or PEG8 molecules but Dex20 and glucose had little effect on binding affinity. Thus, instead of excluded volume effects, the molecules investigated here (both small and large in dimension) may provide other effects (such as soft interactions, potentially impacting protein solvation) that depend on their inherent chemical properties. Such soft interactions, also called nonspecific chemical interactions, are for example hydrophobic, electrostatic or van der Waals interactions that are not strong enough to cause specific binding, but instead are unspecific, rather weak, transient interactions that are dependent on the chemical properties of the respective molecules^42^.

### Crowded environments affect association rate constants for ssDNA binding to *Bs*CspB

To address underlying molecular reasons for altered *K*_D_ (in case of EG, PEG1, or PEG8) or non-altered *K*_D_ values (in case of glucose, or Dex20), we set out to directly probe dissociation rate constants, *k*_off_, and association rate constants, *k*_on_, that together govern the *K*_D_ value, employing fluorescence stopped-flow methodology with millisecond time resolution (Fig. S7A,B). Due to the high viscosity of solution comprising PEG8, we could not use this molecule in these experiments.

**Figure 3:**
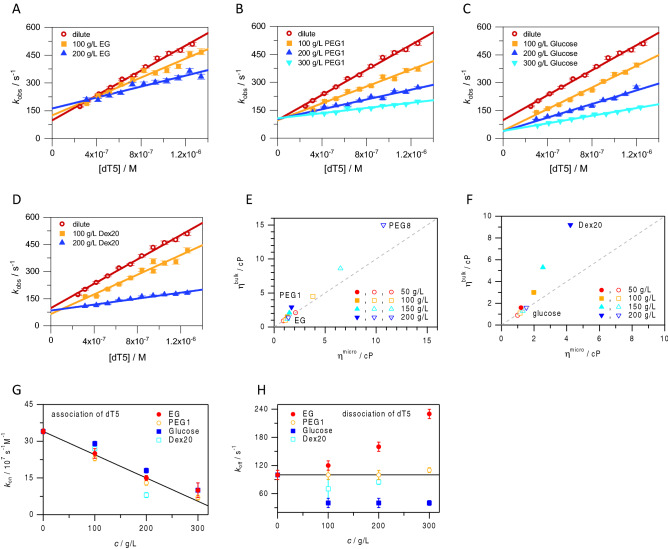
Probing the interaction between the oligothymidine dT5 and *Bs*CspB in absence and presence of a crowded environment by applying kinetic stopped-flow fluorescence spectroscopy considering viscosities of solvents. The apparent rate constant, *k*_obs_, has been obtained for different stoichiometric ratios between dT5 regarding *Bs*CspB under dilute (colored in red, circles) and different concentrations of EG (**A**), PEG1 (**B**), glucose (**C**), and Dex20 (**D**): 100 g/L (colored in orange, rectangles), 200 g/L (colored in blue, triangles with tip to top), and 300 g/L (colored in cyan, triangles with tip to bottom). All experiments have been performed at *T* = 288 K using *c*^*Bs*CspB^ = 60 nM. A linear function has been used to perform regression analysis of experimental data regarding *k*_obs_ (continuous line) to obtain rate constants of ligand association, *k*_on_, and dissociation, *k*_off_. Associated data are presented in Table 2 and Figs. S7A,B and S8A–C. (**E**) Dependence of macro- (η^bulk^) and microviscosity (η^micro^) on the concentration of PEG8 (symbols with central dot), PEG1 (closed symbols), and EG (open symbols). (**F**) Dependence of macro- and microviscosity on the concentration of Dex20 (closed symbols) and glucose (open symbols). The following concentrations of crowding agents have been used: 50 g/L (colored in red, circles), 100 g/L (colored in orange, rectangles), 150 g/L (colored in cyan, triangles with tip to top), and 200 g/L (colored in blue, triangles with tip to bottom). Diffusion coefficient, *D*, probed by NMR spectroscopy reports on the microviscosity of the sample. (**G**, **H**) Kinetic rate constants reporting on association and dissociation of dT5 to *Bs*CspB applying different crowded environments: EG (filled circles, colored in red), PEG1 (open circles, colored in orange), glucose (filled rectangles, colored in blue), Dex20 (open rectangles, colored in cyan). The continuous line represents a linear fit comprising all *k*_on_ values independent of the crowding agent (**G**) as the *k*_off_ value determined under dilute conditions (**H)**.

At dilute conditions, the measured kinetic association rate constants for the interaction of dT5, dT6, and dT7 with *Bs*CspB (Figs. 3A–D, S8A,B, Table 2) match those reported before^34,37^. As the equilibrium affinity increases as the chain length of ssDNA increases (Fig. 2A, Table 1), the dissociation rate constants should stepwise decrease since the association rate constants are similar for dT5, dT6, and dT7 (Table 2). That the reduced binding affinity of shorter oligothymidines to *Bs*CspB in buffer results from an increase of the dissociation rate constant while the association rate constant is maintained is in full agreement with earlier studies^33,34,37^. However, a precise determination of *k*_off_ values binding kinetics of dT6 and dT7 to *Bs*CspB is impeded as the slope defined by the apparent rate constants, *k*_obs_, crosses the ordinate close to zero indicating an extremely slow ligand dissociation in both cases (Fig. S8A,B). Consequently, using the *K*_D_ value obtained in fluorescence experiments in equilibrium before (Table 1) enables an independent analysis of *k*_off_ values characterizing the dissociation of dT6 or dT7 from *Bs*CspB while applying *k*_off_^eq^ = *K*_D_*k_on_. Thus, *k*_off_ = 100 ± 10/s as directly determined for the interaction of dT5 to *Bs*CspB is accompanied by *k*_off_^eq^ = 18 ± 3/s for dT6 and *k*_off_^eq^ = 4 ± 1/s for dT7, respectively (Table 2).

**Table 2:** Numerical values reporting on the association rate constant, *k*_on_, and the dissociation rate constant, *k*_off_, quantifying the interaction between different oligonucleotides and *Bs*CspB obtained under dilute conditions and in crowded environments determined by kinetic stopped-flow fluorescence spectroscopy.

Ligand	Condition	*k*_on_ (10^7^/s/M)	*k*_off_ (/s)	*k*_off_^eq^ (/s)
dT5	Dilute	34 ± 1	100 ± 10	260
dT6	Dilute	32 ± 1	n.d	18
dT7	Dilute	35 ± 4	n.d	4
dT5	100 g/L EG	25 ± 2	120 ± 10	n.d.
dT5	200 g/L EG	15 ± 1	160 ± 10	n.d.
dT5	300 g/L EG	10 ± 3	230 ± 10	510
dT5	100 g/L PEG1	23 ± 1	100 ± 10	n.d.
dT5	200 g/L PEG1	13 ± 1	100 ± 10	n.d.
dT5	300 g/L PEG1	7 ± 1	110 ± 5	270
dT5	100 g/L glucose	29 ± 1	40 ± 10	n.d.
dT5	200 g/L glucose	18 ± 1	40 ± 10	n.d.
dT5	300 g/L glucose	10 ± 3	40 ± 5	130
dT5	100 g/L Dex20	27 ± 2	70 ± 20	190
dT5	200 g/L Dex20	8 ± 1	85 ± 5	50
dT5	300 g/L PEG1	7 ± 1	110 ± 5	266
dT6	300 g/L PEG1	7.8 ± 0.1	10 ± 1	40
dT7	300 g/L PEG1	7.5 ± 0.1	3.1 ± 0.3	13

The value of *k*_off_^eq^ has been calculated by applying *k*_off_^eq^ = *K*_D_*k_on_ (see Table 1 reporting on *K*_D_ values). The concentration of *Bs*CspB was set to 60 nM in all experiments except in presence of 300 g/L EG (*c*^*Bs*CspB^ = 300 nM). A 5- to 20-fold stoichiometric excess of ligand concerning *Bs*CspB has been used in all kinetic stopped-flow fluorescence experiments which have been performed at *T* = 288 K. n.d.: not determined. Associated data are shown in Figs. 3A–D, S7A,B, and S8A–C.

Turning to 300 g/L PEG1 conditions, a prominent impact on the association rate constant is observed for dT5, dT6, and dT7 (Figs. 3B, S8A,B, Table 2). The association rate constant decreases coherently by about fivefold for the three different oligonucleotides dT5, dT6, and dT7 comparing dilute conditions (*k*_on_ ≈ 34 ± 2 × 10^7^/s/M) with an environment constituted by 300 g/L PEG1 (*k*_on_ ≈ 7.4 ± 0.4 × 10^7^/s/M, Table 2). Note that as in experiments performed in pure buffer, the association rate constants in PEG1 are independent of the length of the oligothymidine (Table 2). Also, dissociation kinetics of the interaction between oligothymidines and *Bs*CspB that are performed in presence of PEG1 follows the same trend as found in pure buffer. Addition of 300 g/L PEG1 leads to dissociation rate constants which decrease gradually starting from *k*_off_ = 110 ± 5/s (observed for dT5), to *k*_off_ = 10 ± 1/s (observed for dT6) and finally to *k*_off_ = 3.1 ± 0.3/s (observed for dT7) (Fig. S8A,B, Table 2). Focusing on the interaction of dT5 with *Bs*CspB we probed the effect of varying concentration of the added molecule. However, *k*_off_ values are almost identical in presence of 100 g/L PEG1, 200 g/L PEG1, and 300 g/L PEG1 (Fig. 3B, Table 2). For Dex20, like for PEG1, there was a slowing effect on *k*_on_ value when Dex20 concentration was increased but no significant effect on *k*_off_ values were observed (Fig. 3D, Table 2). Note that *k*_on_ values found for binding of dT5 to *Bs*CspB in presence of 100 g/L PEG1 or 200 g/L PEG1 (Fig. 3B) are comparable to *k*_on_ values found in presence of identical concentrations of Dex20 (Fig. 3D, Table 2). It has to be considered that different viscosities present in solutions that are largely supplemented by inert molecules may affect molecular diffusion, and thus eventually the kinetics of ligand binding. Note that the bulk viscosity of solutions comprising 200 g/L EG, 200 g/L PEG1, or 200 g/L PEG8 covers considerably more than one order of magnitude (Fig. 3E). In parallel, the microviscosity changes only by about one order of magnitude when turning from EG to PEG8 applying the same concentration (Fig. 3E). The same observation is made for glucose and Dex20 (Fig. 3F). Differences in the viscosity of the solvent used (e.g. a ratio of about 3 comparing bulk viscosities of 200 g/L Dex20 with 200 g/L PEG1 and of about 2.5 comparing corresponding microviscosities) do not per se result in *k*_on_ values of pronounced differences characterizing the interaction of dT5 with *Bs*CspB. Thus, an identical concentration of molecules that are added to the solution under study leads to comparable kinetics of ligand association whereas ligand dissociation shows differences between solutions comprising PEG1 or Dex20.

In a next step, we extended the kinetic analysis to the interaction between ssDNA and *Bs*CspB in the presence of low molecular weight EG and glucose. The *k*_on_ values determined in presence of EG or glucose agree quantitatively very well with the findings made in presence of PEG1 or Dex20 before. The association rate constant characterizing binding of dT5 to *Bs*CspB decreases with increasing concentration of EG (Figs. 2A, S8C) or glucose (Fig. 2C, Table 2). Thus, this general trend cannot be solely explained by viscosity effects as macro- and microviscosities for solutions comprising EG, PEG1, glucose, or Dex20 molecules vary significantly (Fig. 3E,F). However, since equilibrium affinity, the *K*_D_ value, was affected differently by EG and glucose (Fig. 2C, Table 1), we anticipated differences in *k*_off_ values. Indeed, the dissociation of dT5 from *Bs*CspB is decreased by a factor of about two to three in presence of 300 g/L glucose as compared to dilute conditions, whereas 300 g/L EG increases *k*_off_ by up to two-fold (Fig. 3H, Table 2). The effect of EG was also tested at different concentrations: for 100 g/L, to 200 g/L, and 300 g/L EG, the observed *k*_off_ value raises gradually (Figs. 3A, S8C, Table 2). Contrarily, this is not observed when the concentration of glucose increases: the *k*_off_ value remains at 40/s for 100 g/L, 200 g/L, and 300 g/L glucose (Fig. 3H, Table 2).

Summing up, all four molecules supplemented to the solution that is studied in kinetic experiments, PEG1, Dex20, EG, and glucose, slow down association rate constants in a concentration-dependent manner (Fig. 3G). However, whereas larger molecules PEG1 and Dex20 have no significant effect on *k*_off_ rate constants, low molecular weight EG and glucose affect that parameter, too (Fig. 3H). While EG increases *k*_off_ rate constants, the presence of glucose is capable to decrease *k*_off_ rate constants. The differential modulation of kinetic rate constants observed for the utilization of different crowding agents implies that altered viscosity of the solvent (on both microscopic and macroscopic scale) is not responsible for this observation. Also effects due to excluded volume caused by the molecules which have been added to the solution under study, including depletion interactions, cannot exclusively explain our experimental data.

### Solvent exchange of *Bs*CspB amide protons depends on particular crowded environment in use

**Figure 4:**
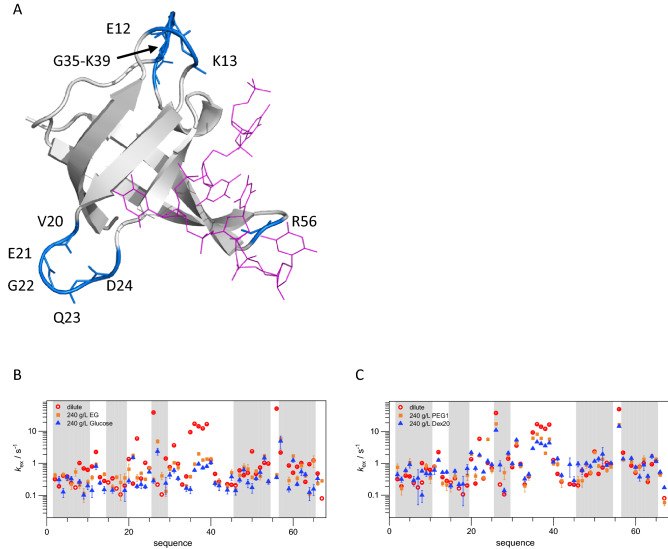
Impact of the presence of a crowded environment on the exchange between amide protons comprising *Bs*CspB with solvent protons as probed by NMR spectroscopy. (**A**) The backbone of *Bs*CspB is shown in cartoon mode (colored in gray) whereas dT6 is shown by using lines (colored in pink). Amide protons comprising *Bs*CspB which are highly affected upon adding of 240 g/L EG (colored in orange, rectangles, (**B**)), 240 g/L glucose (colored in blue, triangles, (**B**)) as well as 240 g/L PEG1 (colored in orange, rectangles, (**C**)) or 240 g/L Dex20 (colored in blue, triangles, (**C**)) in comparison to dilute conditions (colored in red, circles, (**B**) and (**C**)) are highlighted by showing main chain atoms in stick mode (colored in blue). Associated data presenting residue specific buildup curves is shown in Fig. S9A–J. This figure has been prepared using 2ES2.pdb and PyMol (www.pymol.org).

The kinetic analysis showed that the change in association kinetics between ssDNA and *Bs*CspB does not depend on the type or size of the molecule that is added to the solution under study. In contrast, the change in dissociation kinetics depends on the specific choice of this molecule (Table 2): molecules possessing low molecular weight affect *k*_off_ rate constants (showing different directions for EG and glucose) but larger molecules had no effect on *k*_off_ rate constants. To address these puzzling results, that potentially hint towards solvent interactions and dynamics, we set out to probe the hydration shell around the protein as a function of the molecule which is added. Effects on water exchange dynamics due to presenting crowding environments may in turn modify the interaction between ssDNA and *Bs*CspB (Fig. 4A–C). Note that a modified MEXICO approach was successfully applied before in order to explain the increase in overall thermodynamic stability of *Bs*CspB at a residue-by-residue level when dilute conditions and crowded environments (comprising Dex20, or PEG8 molecules) were compared^26^. Here, we systematically probed solutions of 240 g/L EG, 240 g/L glucose, 240 g/L PEG1, or 240 g/L Dex20 for their effects on the exchange of water protons with individual amide protons in *Bs*CspB and compared to dilute conditions (Fig. S9A–J). The average of the rate constant of exchange for all residues which could be followed, *k*_ex_^all^, was specified to *k*_ex_^all,dilute^ = (3.2 ± 8.5)/s (Fig. 4B,C). Interestingly, the region of amino acids possessing largest changes in chemical shifts upon interaction with dT7 (CSP ≃ 0.5 ppm seen for F38, K39, T40) determined under dilute conditions (Fig. S1C) and crowded environments (Fig. S1D,E) exhibit highest values in the exchange rate constant with *k*_ex_^G35-K39,dilute^ ≃ 10.5/s, too (Fig. 4B,C). Focusing on crowded environments, *c*^glucose^ = 240 g/L induces the strongest decrease in *k*_ex_^all^ values compared to dilute conditions: *k*_ex_^all,240g/Lglucose^ = (0.5 ± 0.7)/s (Fig. 4B). This also holds for the analysis of the particular region which is predominantly affected by interaction with ssDNA: *k*_ex_^G35-K39,240g/Lglucose^ ≃ 1/s (Fig. 4B). The slower water exchange dynamics correlate with slower *k*_off_ values (Table 2) at this condition. In contrast to glucose, EG increases *k*_off_ values (Table 2) and in the presence of *c*^EG^ = 240 g/L the *k*_ex_ value of almost all residues is higher for *c*^EG^ = 240 g/L compared to *c*^glucose^ = 240 g/L which leads to *k*_ex_^all,240g/LEG^ = (0.7 ± 1.0)/s and *k*_ex_^G35-K39,240g/LEG^ ≃ 1.5/s (Fig. 4B), respectively. To complement, *c*^Dex20^ = 240 g/L leads to *k*_ex_^all,240g/LDex20^ = 1.5 ± 2.4/s whereas *c*^PEG1^ = 240 g/L leads to *k*_ex_^all,240g/LPEG1^ = 2 ± 3/s (Fig. 4C). The same tendency is observed when analyzing the region of largest changes of chemical shifts upon interaction of *Bs*CspB with ssDNA: *k*_ex_^G35-K39,240g/LDex20^ ≃ 4.5/s compared to *k*_ex_^G35-K39,240g/LPEG1^ ≃ 7/s (Fig. 4C). For all crowded environments, the exchange between amide and solvent protons is slower than at dilute conditions, but to various degrees. We note that *k*_ex_ values observed for amide protons in *Bs*CspB decrease by about 30 percent when turning from *c*^EG^ = 240 g/L to *c*^glucose^ = 240 g/L conditions. The same decrease in *k*_ex_ values is observed when turning from *c*^PEG1^ = 240 g/L to *c*^Dex20^ = 240 g/L conditions. The analysis of exchange between amide and solvent protons suggests effects on dynamics in the water hydration shell encompassing *Bs*CspB but cannot give full explanations for the *k*_off_ values observed in kinetic stopped-flow experiments (Table 2). However, the changes in *k*_ex_ values observed when comparing the effects of low molecular weight glucose with EG and larger Dex20 with PEG1 are consistent with the trends in dissociation rate constants that were independently obtained by fluorescence stopped-flow methodology. Thus, the analysis of amide to solvent proton exchange experiments conducted at atomic resolution hints at modifications of the hydration shell around *Bs*CspB as the source of cosolvent induced effects on protein–DNA binding. In addition, we found that neither *c*^EG^ = 200 g/L nor *c*^glucose^ = 200 g/L induces changes in NMR chemical shifts of the aromatic side chains (Fig. S10A,B) which constitute the main binding site of DNA in *Bs*CspB. Importantly, none of our data suggest direct interactions of low molecular weight subunits (EG, glucose) and macromolecular crowders (PEG1, Dex20) with the protein. Instead, the observed effects appear mediated indirectly (hydration effects) or via weak, transient interactions that are dependent on the chemical features of the particular molecule in use.

## Discussion

To investigate the impact of crowded environments on protein-to-DNA interactions, the binding of oligothymidines of variable chain length to *Bs*CspB were systematically examined by a variety of biophysical approaches. To create crowded environments, increasing weight volume fractions of low molecular weight molecules as well as macromolecules were included and experimental data was acquired at global as well as at atomic resolution. To be able to specifically compare size as well as chemical effects, we consequently used both small and large molecules of two types: EG, PEG1 and PEG8 as well as glucose and Dex20. First, upon analyzing the chemical shift for proton, carbon, nitrogen, and phosphorous resonance signals by high resolution NMR spectroscopy we revealed complete structural integrity of free and ligand-bound states of *Bs*CspB in presence of the different crowded environments. Thus, no significant perturbation of the common binding site between ssDNA and *Bs*CspB^36^ could be observed at these conditions. However, differences in signal heights and line widths of NMR resonance signals comprising *Bs*CspB hinted to specific changes in the dynamics of the protein-to-ligand interaction when comparing dilute conditions with crowded environments. This observation was corroborated by a precise analysis performed at a residue-by-residue level. Notably, residue Arg56 interacts with the phosphate backbone of oligonucleotides^36^ and the inherent flexibility of the loop region where Arg56 is positioned is essential for high affinity binding of ssDNA to *Bs*CspB^34^. We found effects of crowding on cross-peak signal height for Arg56, and the amide proton of Arg56 was one of the most affected when comparing exchange with solvent protons under dilute versus crowded conditions (Figs. 4A,B, S9J).

In terms of thermodynamic aspects of the DNA–protein interaction, we found a significant decrease in ligand binding affinity when comparing dilute conditions with crowded environments of EG, PEG1, or PEG8 (Figs. 2A,C,D, S6A–D). This observed decrease in ligand binding affinity is strongly dependent on the weight per volume fraction of the molecules present in solution, but not the molecular size of the particular molecule being used (Fig. 2D, Table 1). The magnitude of this effect is dependent on the length of the oligothymidine in use. An about 20-fold decrease in binding affinity to *Bs*CspB was observed comparing dilute conditions with the presence of *c*^PEG1^ = 300 g/L for dT7 (Fig. S6A,B), which possesses an affinity in the low nanomolar range under dilute conditions, but, only a three-fold decrease in affinity when comparing dilute conditions with *c*^PEG1^ = 300 g/L for dT4 (Fig. 2A), an oligonucleotide possessing a binding affinity to *Bs*CspB in the micromolar range under dilute conditions. Notably, in contrast to EG, PEG1 and PEG8, solutions comprising glucose or Dex20 at about 300 g/L are not capable to significantly modify the binding affinity of dT5 or dT7 to *Bs*CspB when compared to dilute conditions (Figs. 2B–D, S6E).

To understand the underlying reasons for the differential effects in ligand binding affinity, we explored ligand association and complex dissociation kinetics at different conditions. These experiments revealed that a crowded environment is generally capable to significantly decrease the association rate constant as a function of the concentration of crowding agents (Fig. S3G). The observed change in the association rate constant is possibly mediated by a crowding agent induced reduction of structural flexibility in the regions carrying residues Lys7, Asp25, and Arg56 comprising *Bs*CspB, which exert long range charge-charge interactions^36^ and are in part located at sites of great conformational flexibility under dilute conditions, but do not possess significant changes in chemical shifts upon ligand interaction (Fig. S1C). Long range coulombic interactions have been identified to initiate protein–ligand interaction^43^ and therefore the association rate constant might inherently depend on the flexibility of regions carrying charged residues that allow ligand recognition and binding. In contrast to effects seen for the rate constant of ligand association, the change in the dissociation rate constant is dependent on the particular molecule that is added to the solution under study (Fig. 3H). As compared to dilute conditions, the presence of EG increased the dissociation rate constant in a concentration-dependent manner. On the other hand, PEG1, glucose and Dex20 did not show this concentration dependent increase: PEG1 did not affect dissociation rate constant at all as compared to dilute conditions, for glucose, the dissociation rate constants were roughly halved as compared to in buffer only. The presence of Dex20 slowed down ligand dissociation to a value in between that was found for glucose and that for applying dilute conditions. Taken together, the decrease in DNA–protein affinity found for EG and PEG1 (Fig. 2E) arises from slower ligand association only (in the case of PEG1) and from slower association combined with faster dissociation (in the case of EG). For Dex20 and glucose, the almost invariant ligand binding affinity (Fig. 2E) is due to compensating effects on the kinetic rate constants, such that slower ligand association is paired with slower ligand dissociation. Here the effect on ligand association is larger for Dex20, but the effect on dissociation is larger in magnitude for glucose.

The results open several questions to be answered before there is quantitative understanding of the observations: why are all association rate constants slowed down in a similar manner for all different molecules that have been added to the solution under study when the micro- and macroviscosity differ among these conditions? Why is the dissociation of ssDNA from *Bs*CspB accelerated when EG (but no other molecule) is used? And why does glucose and Dex20 retard ligand dissociation, but this is not seen for EG and related molecules with increasing degree of polymerization? Finally, what is the role of excluded volume effects in the experimental data that have been obtained in presence of PEG and Dex20?

We propose that inherent chemical features of the molecule under study determine the precise impact on ligand-to-protein affinity via the molecules’ differential ability to manipulate the protein’s hydration shell. Thus, we propose that low molecular weight EG modifies the DNA binding interface in *Bs*CspB such that it promotes faster DNA dissociation. EG may be incorporated into the hydration shell around *Bs*CspB due to its inherent ability of solvation^44,45^: this small organic compound and its polymeric derivatives harbor to some extent hydrophobic characteristics similar to a weak organic solvent. In contrast, glucose tends to be excluded from *Bs*CspB’s hydration shells due to its more polar character^46–49^. In accord with opposite behavior, glucose does not increase the dissociation rate constant but instead slows it down. These effects become less pronounced for the larger molecules PEG1 and Dex20 as the number of molecules that are added to the solution of interest decrease with increasing molecular weight while the concentration in mass is kept constant. Additionally, chemical features as e.g. polarity and capability of hydrogen bonding are not identical when comparing larger molecules with monomeric counterparts while keeping concentration in mass constant. Using a battery of NMR probes including ^1^H, ^13^C, ^15^N, and ^31^P chemical shifts for *Bs*CspB and the ligand has not given experimental evidence for direct interactions with any of the molecules used here for supplementation. Instead, our data exclude such distinct direct interactions. Therefore, we argue that ‘soft’ interactions between the molecule added to the solution (with regard to its chemistry) and the hydration shell encompassing the protein regulate ligand binding affinity via modulation of both association and dissociation kinetics. Such molecule-dependent modification of protein hydration has been reported before^50,51^. Especially, hydration effects were used to explain the slowing down of water dynamics around a protein in presence of PEG while the hydrogen bonding network was still unaffected^52^. It is important to note that it was not possible to unravel these ‘soft’ interactions in our experiments even if we performed careful, multidimensional analyses of the chemical environment of the protein and the ligand at atomic resolution. Essentially, the combined application of dynamic NMR and kinetic stopped-flow fluorescence experiments was required to consistently interpret the underlying mechanism governing the impact each crowded environment had on the DNA–protein system. Importantly, the profound and differential effects observed here cannot be explained by excluded volume theory which would predict same effects for all inert (macro)molecules. Nonetheless, there will be some excluded volume in presence of PEG1 and Dex20 molecules, that is absent in presence of EG and glucose, that may provide steric effects as an additional but not dominant factor.

To sum up, we conclude that non-dilute environments can have substantial impact on the association/dissociation kinetics between nucleic acid ligands and target proteins via hydration shell mediated modifications of soft interactions. This result questions studies solely performed in vitro under dilute conditions regarding the quantitative determination of ligand binding affinity and kinetics. It has to be expected that binding reactions monitored in vitro are not necessarily indicative of the true affinity or ligand specificity in vivo*.* A crowded environment is inherently capable to impact intrinsic dynamics of proteins and interactions among them via excluded volume (*steric*, dominant for large molecules), depletion (*attractive*, based on exclusion of solutes from the proximity of inert particles) and solvent (*soft interactions*) effects. This may especially affect dynamics at surface exposed motile loop regions^50^, areas in a protein which are easily accessible for solvent^26^, and long range charge–charge interactions between the ligand and the target protein (if the added molecule possesses charge). Note that we have applied here crowded environments possessing concentrations which are below (true for EG and glucose) and above (true for PEG1 and Dex20) a critical concentration, c*, that separates between a dilute and a semi-dilute regime of the molecules that are used to crowd the solution under study^17^. However, the impact such solutions have on the affinity between dT5 and *Bs*CspB are comparable for EG (below c*) and PEG1 (above c*) as well as for glucose (below c*) and Dex20 (above c*) (Fig. 2C,D) eventhough they individually represent different regimes. Thus, our study shows that for the DNA–protein system comprehensively investigated here, soft interactions—that are dependent on the chemical nature of the surrounding molecule added in high concentration—may dominate.

Taken together, crowded environments have intricate effects on ligand-to-protein binding processes and may tune, in nontrivial and chemistry-specific manners involving both entropic and enthalpic forces, many key reactions in vivo, for example signal transduction which often involves transient ligand–protein interactions.

## Material and methods

### Protein expression and purification

Protein expression and purification has been conducted as described previously^26^. Purified protein samples were shock frozen and stored at − 80 °C.

### NMR measurements

All samples were probed at *T* = 298 K or *T* = 294.4 K and contained 5% v/v D_2_O, 20 mM disodium phosphate at pH 7, and variable amounts of polyethylene glycol of a molecular weight of 1 kDa (PEG1, purchased from Roth), 8 kDa (PEG8, purchased from Roth), or dextran of a molecular weight of 20 kDa (Dex20, purchased from Pharmacosmos). Oligonucleotide to *Bs*CspB titrations started at a protein concentration of 250 µM for dilute conditions and 100 g/L PEG8, and at a protein concentration of 150 µM for dilute conditions, 200 g/L PEG8, and 300 g/L PEG1, and 300 g/L Dex20. The stepwise addition of heptathymidine (purchased from biomers) up to a twofold excess regarding concentration of *Bs*CspB leads to a final increase in sample volume of < 5%. Two-dimensional ^1^H–^15^N HSQC and ^1^H–^13^C HSQC NMR spectra as well as one dimensional ^31^P spectra were recorded on a Bruker Neo 800 MHz spectrometer equipped with a cryogenic probe head or on a Bruker Avance III 600 MHz spectrometer also equipped with a cryogenic probe head. Readout of cross-peak signal heights and chemical shifts occurred using the software nmrView^53^, the spectra were processed using the software nmrPipe^54^. Readout of line width of cross-peaks occurred by the command “peakw” using the software Topspin (Bruker). The assignment of backbone ^1^H–^15^N resonance signals of the free state of *Bs*CspB has been transferred from Balbach et al.^33^ and has been verified by acquiring HNCA, HNCAB, and HN(CO)CACB experiments^55^. The assignment of resonance signals of the DNA-bound state of *Bs*CspB has been adapted from Zeeb et al.^34^. The assignment of resonance signals reporting on aromatic residues comprising *Bs*CspB acquired in 2D ^1^H–^13^C HSQC spectra have been obtained from Weininger et al.^56^. Triple-resonance NMR experiments were conducted on a Bruker Avance III 600 MHz spectrometer equipped with a cryogenic probe head. All spectra were directly referenced in the proton dimension to TMSP (purchased from Alfa Aesar) that was added to each sample to a final concentration of 70 µM.

### Analysis of chemical shift perturbations (CSPs)

For the calculation of CSPs, ^1^H–^15^N HSQC spectra of 150 µM *Bs*CspB (20 mM disodium phosphate/HCl, pH 7.0) in absence or in presence of 300 g/L PEG1 were acquired on a Bruker 800 MHz spectrometer equipped with a cryogenic probe head operated at *T* = 298 K, spectra of *Bs*CspB in presence of 300 g/L Dex20 were acquired on a Bruker Avance III 600 MHz spectrometer equipped with a cryogenic probe head operated at *T* = 298 K. All spectra were referenced to TMSP (^1^H dimension) as well as to ^15^N ammonium chloride (^15^N dimension) under the respective conditions. Perturbations of chemical shifts were weighted according to Grzesiek et al.^57^.

### Analysis of rate constants applying amide to solvent proton exchange methodology

Two-dimensional heteronuclear ^1^H–^15^N NMR spectra were acquired for *Bs*CspB on a Bruker Avance III 600 MHz spectrometer equipped with a cryogenic probe head operated at *T* = 298 K using a modified MEXICO pulse sequence (Measurement of EXchange rates in Isotopically labeled COmpounds)^58^ using mixing times of *t*_mix_ = 10, 20, 30, 50, 70, 80, 100, 120, 140, 160, 180, 200 and 250 ms in absence and presence of *c*^EG^ = 240 g/L, *c*^PEG1^ = 240 g/L, *c*^glucose^ = 240 g/L, or *c*^Dex20^ = 240 g/L. Double measurements of mixing times of *t*_mix_ = 30, 100, and 200 ms were applied to account for the experimental error. Regression of experimental data was done according to:

1 $${I}_{\text{ref}}=\frac{{k}_{\text{ex}}}{{R}_{\text{1NH}}+{k}_{\text{ex}}-{R}_{\text{1W}}}*(\text{exp}\left(-{R}_{\text{1W}}*{t}_{\text{mix}}\right)-\text{exp}\left(-\left({R}_{\text{1NH}}+{k}_{\text{ex}}\right)*{t}_{\text{mix}}\right),$$

where *I*_ref_ is the intensity in the modified MEXICO HSQC spectrum referenced to a ^1^H–^15^N HSQC spectrum omitting a mixing period, *R*_1NH_ is the longitudinal relaxation rate constant of the respective amide proton, *R*_1W_ is the longitudinal relaxation rate constant of water and *k*_ex_ the exchange rate constant of the amide proton with solvent protons. The longitudinal relaxation rate constant of water was independently obtained by an inversion recovery experiment and yielded *R*_1W_ = 0.29 ± 0.01/s (dilute conditions), *R*_1W_ = 0.45 ± 0.01/s (240 g/L Dex20), *R*_1W_ = 0.45 ± 0.01/s (240 g/L PEG1), *R*_1W_ = 0.49 ± 0.01/s (240 g/L glucose), and *R*_1W_ = 0.51 ± 0.01/s (240 g/L EG). The NMR samples contained about *c*^*Bs*CspB^ = 0.5 mM. Data regression occurred using the software OriginLab.

### Determination of macro- and microviscosity

Macroviscosities, η^bulk^, of solutions containing different concentrations of EG, PEG1, glucose, or Dex20 molecules have been determined at *T* = 298 K by using a Viscolite 700 d15 instrument (precision of ± 0.1 cP). Corresponding microviscosities have been determined on a Bruker Avance III 600 MHz spectrometer equipped with a cryogenic probe head at *T* = 298 K by applying diffusion NMR methodology. Therefore, a pulse sequence comprising a stimulated echo assisted by bipolar gradients, *G*, employing an adapted diffusion time, Δ, ranging between 30 and 120 ms, and a gradient length, δ, of 3 ms along the z-axis was applied^59^. Gradients were calibrated as described in^60^. Three different gradient strengths have been repeated three times each to estimate the experimental error. Integrals for proton signals, *I*, were determined for EG, PEG1, glucose, and Dex20 and used for calculation of the diffusion coefficient, *D*:

2 $$I\left( G \right) = I\left( 0 \right){\text{exp}}( - G^{{2}} \gamma^{{2}} \delta^{{2}} D(\Delta - \delta /{3})),$$

where γ is the gyromagnetic ratio of protons. Data regression occurred using the software OriginLab. According to the Stokes–Einstein equation, *D* is a direct measure for the microviscosity, η^micro^, in the respective sample comprising different concentrations of EG, PEG1, glucose, or Dex20 molecules. The following hydrodynamic radii, *r*_H_, have been used to finally determine η^micro^: *r*_H_^EG^ = 2.9 Å^61^, *r*_H_^glucose^ = 4.5 Å^62^, *r*_H_^PEG1^ = 9.4 Å^61^, *r*_H_^Dex20^ = 32.4 Å^63^.

### Steady state fluorescence measurements

Thermally equilibrated samples were measured under stirring in a temperate-regulated holder at *T* = 298 K, using a silica glass cuvette of 10 mm diameter (Helma). An FP-8500 spectrofluorometer (Jasco) was used for excitation of intrinsic tryptophan of *Bs*CspB at a wavelength of 280 nm, detection of fluorescence emission spectra occurred from 290 to 400 nm. Fluorescence intensity readout occurred at a wavelength of 342 nm. All samples contained 20 mM disodium phosphate at pH 7, and variable amounts of ethylene glycol (EG, purchased from Roth), polyethylene glycol of a molecular weight of 1 kDa (PEG1, purchased from Roth) or 8 kDa (PEG8, purchased from Roth), glucose (purchased from Sigma Aldrich), or dextran of a molecular weight of 20 kDa (Dex20, purchased from Pharmacosmos). Titration experiments started at a protein concentration of 4 µM, 2.8 µM, or 0.5 µM. The stepwise addition of the respective oligonucleotide (purchased from biomers) to at least a five-fold excess regarding *Bs*CspB caused a final increase in sample volume of < 3%. For each titration experiment, at least 28 measuring points were recorded where each measuring point is the average of triplicate measurements. The concentration of nucleic acids was determined via absorbance measurement using a NanoDrop One setup (Thermo Fisher Scientific) with the specific extinction coefficients ε_260_^dT4^ = 36,800/M/cm, ε_260_^dT5^ = 46,000/M/cm, ε_260_^dT6^ = 55,200/M/cm, and ε_260_^dT7^ = 64,400/M/cm. In respect to the buffer’s background fluorescence, all raw data were corrected by data regression of blank measurements with increasing concentrations of each oligo nucleotide under the respective buffer condition. Analysis of steady state fluorescence data occurred according to the binding equation^64,65^:

3 $$Q={Q}_{\text{max}}\times \frac{\sqrt{{A}^{2}-4n\times {\left[P\right]}_{0}\times {\left[L\right]}_{0}}}{2\times {\left[P\right]}_{0}},$$

with$$:$$

$$A={K}_{\text{D}}+{\left[P\right]}_{0}+n\times {\left[L\right]}_{0,}$$

where *Q* stands for quenching of intrinsic tryptophan fluorescence upon addition of the respective nucleotide. *Q*_max_ represents the maximal quenching observed upon saturation of the binding event in *Bs*CspB using respective ssDNA. [*P*]_0_ and [*L*]_0_ are the overall concentrations of protein and ligand, *n* is the stoichiometric binding factor of the protein–DNA complex, and *K*_D_ is the dissociation constant of the complex. Data regression occurred using the software OriginLab.

### Kinetic fluorescence measurements

Kinetic fluorescence measurements were conducted using an SX-20 stopped flow machine (Applied Photophysics). Excitation occurred at a wavelength of 278 nm, emission was monitored using a 320 nm cut-off filter. All experiments were conducted at *T* = 288 K using *c* = 60 nM or *c* = 300 nM (holds for *c*^EG^ = 300 g/L only) *Bs*CspB in 20 mM disodium phosphate buffer, pH 7.0 and variable amounts of ethylene glycol (EG, purchased from Roth), polyethylene glycol of a molecular weight of 1 kDa (PEG1, purchased from Roth), glucose (purchased from Sigma Aldrich), or dextran of a molecular weight of 20 kDa (Dex20, purchased from Pharmacosmos) to implement a crowded environment while using a 5 to 20-fold excess of oligothymidines (purchased from biomers) regarding concentration of *Bs*CspB. This experimental setup allows to apply a pseudo-first order approach for data evaluation^33^. Thus, linear regression of the observed rate constant, *k*_obs_, plotted over the concentration of ligand provides ligand association, *k*_on_, as slope and the dissociation rate constant, *k*_off_, as interception with the y-axis. A more accurate estimation for *k*_off_ is further derived from the relation *k*_off_^eq^ = *k*_on_*K_D_, where *K*_D_ has been estimated by fluorescence experiments in equilibrium (see Eq. 3). The oligonucleotides dT5, dT6, and dT7 were used in kinetic experiments following the interaction with *Bs*CspB.

## Supplementary Information

Supplementary Figures.

## Data Availability

The data that support the findings of this study are available from the corresponding author upon reasonable request. Source data are provided with this paper.

## References

[1] Cayley S, Lewis BA, Guttman HJ, Record MT (1991). Characterization of the cytoplasm of *Escherichia coli* K-12 as a function of external osmolarity: Implications for protein–DNA interactions in vivo. J. Mol. Biol..

[2] Zimmerman SB, Trach SO (1991). Estimation of macromolecule concentrations and excluded volume effects for the cytoplasm of *Escherichia coli*. J. Mol. Biol..

[3] Damman R (2019). Atomic-level insight into mRNA processing bodies by combining solid and solution-state NMR spectroscopy. Nat. Commun..

[4] Brangwynne CP, Tompa P, Pappu RV (2015). Polymer physics of intracellular phase transitions. Nat. Phys..

[5] An S, Kumar R, Sheets ED, Benkovic SJ (2008). Reversible compartmentalization of de novo purine biosynthetic complexes in living cells. Science.

[6] Aragón JJ, Sols A (1991). Regulation of enzyme activity in the cell: Effect of enzyme concentration. FASEB J..

[7] Puchkov EO (2013). Intracellular viscosity: Methods of measurement and role in metabolism. Biochem. (Moscow) Suppl. Series A Membrane Cell Biol..

[8] Kuhn W (1934). Über die Gestalt fadenförmiger Moleküle in Lösungen. Kolloid-Zeitschrift.

[9] Flory PJ (1949). The configuration of real polymer chains. J. Chem. Phys..

[10] Minton AP, Wilf J (1981). Effect of macromolecular crowding upon the structure and function of an enzyme: Glyceraldehyde-3-phosphate dehydrogenase. Biochemistry.

[11] Minton AP (2006). Macromolecular crowding. Curr. Biol..

[12] Minton AP (2017). Explicit incorporation of hard and soft protein–protein interactions into models for crowding effects in protein mixtures. II. Effects of varying hard and soft interactions upon prototypical chemical equilibria. J. Phys. Chem. B.

[13] Sharp KA (2015). Analysis of the size dependence of macromolecular crowding shows that smaller is better. Proc. Natl. Acad. Sci..

[14] Parsegian VA, Rand RP, Rau DC (2000). Osmotic stress, crowding, preferential hydration, and binding: A comparison of perspectives. Proc. Natl. Acad. Sci..

[15] Minton AP (1981). Excluded volume as a determinant of macromolecular structure and reactivity. Biopolymers.

[16] Zhou HX, Rivas G, Minton AP (2008). Macromolecular crowding and confinement: biochemical, biophysical, and potential physiological consequences. Annu. Rev. Biophys..

[17] Rivas G, Minton AP (2018). Toward an understanding of biochemical equilibria within living cells. Biophys. Rev..

[18] Somalinga BR, Roy RP (2002). Volume exclusion effect as a driving force for reverse proteolysis: Implications for polypeptide assemblage in a macromolecular Crowded Millieu. J. Biol. Chem..

[19] Herzfeld J (2004). Crowding-induced organization in cells: Spontaneous alignment and sorting of filaments with physiological control points. J. Mol. Recognit..

[20] Zosel F, Soranno A, Buholzer KJ, Nettels D, Schuler B (2002). Depletion interactions modulate the binding between disordered proteins in crowded environments. Proc. Natl. Acad. Sci..

[21] Schellman JA (2003). Protein stability in mixed solvents: A balance of contact interaction and excluded volume. Biophys. J..

[22] Hoppe T, Minton AP (2019). Non-specific interactions between macromolecular solutes in concentrated solution: Physico-chemical manifestations and biochemical consequences. Front. Mol. Biosci..

[23] Feng B (2019). Hydrophobic catalysis and a potential biological role of DNA unstacking induced by environment effects. Proc. Natl. Acad. Sci..

[24] Schnell S, Turner TE (2004). Reaction kinetics in intracellular environments with macromolecular crowding: Simulations and rate laws. Prog. Biophys. Mol. Biol..

[25] Kozer N, Kuttner YY, Haran G, Schreiber G (2007). Protein–protein association in polymer solutions: From dilute to semidilute to concentrated. Biophys. J..

[26] Köhn B, Kovermann M (2019). Macromolecular crowding tunes protein stability by manipulating solvent accessibility. ChemBioChem.

[27] Köhn B, Kovermann M (2020). All atom insights into the impact of crowded environments on protein stability by NMR spectroscopy. Nat. Commun..

[28] Wang Y, Sarkar M, Smith AE, Krois AS, Pielak GJ (2012). Macromolecular crowding and protein stability. J. Am. Chem. Soc..

[29] Soranno A (2014). Single-molecule spectroscopy reveals polymer effects of disordered proteins in crowded environments. Proc. Natl. Acad. Sci. USA.

[30] Schindelin H, Marahiel MA, Heinemann U (1993). Universal nucleic acid-binding domain revealed by crystal structure of the *B. subtilis* major cold-shock protein. Nature.

[31] Schnuchel A (1993). Structure in solution of the major cold-shock protein from *Bacillus subtilis*. Nature.

[32] Lopez MM, Yutani K, Makhatadze GI (1999). Interactions of the major cold shock protein of *Bacillus subtilis* CspB with single-stranded DNA templates of different base composition. J. Biol. Chem..

[33] Zeeb M, Balbach J (2003). Single-stranded DNA binding of the cold-shock protein CspB from *Bacillus subtilis*: NMR mapping and mutational characterization. Protein Sci..

[34] Zeeb M (2006). Recognition of T-rich single-stranded DNA by the cold shock protein Bs-CspB in solution. Nucleic Acids Res..

[35] Max KE, Zeeb M, Bienert R, Balbach J, Heinemann U (2006). T-rich DNA single strands bind to a preformed site on the bacterial cold shock protein Bs-CspB. J. Mol. Biol..

[36] Max KEA, Zeeb M, Bienert R, Balbach J, Heinemann U (2007). Common mode of DNA binding to cold shock domains. FEBS J..

[37] Sachs R, Max KE, Heinemann U, Balbach J (2012). RNA single strands bind to a conserved surface of the major cold shock protein in crystals and solution. RNA.

[38] Johnson DS, Mortazavi A, Myers RM, Wold B (2007). Genome-wide mapping of in vivo protein–DNA interactions. Science.

[39] Steitz TA (1990). Structural studies of protein–nucleic acid interaction: The sources of sequence-specific binding. Q. Rev. Biophys..

[40] von Hippel PH, Bear DG, Morgan WD, McSwiggen JA (1984). Protein–nucleic acid interactions in transcription: A molecular analysis. Annu. Rev. Biochem..

[41] Kleckner IR, Foster MP (2011). An introduction to NMR-based approaches for measuring protein dynamics. Biochimica et Biophysica Acta (BBA) Proteins Proteom..

[42] Kuznetsova I, Zaslavsky B, Breydo L, Turoverov K, Uversky V (2015). Beyond the excluded volume effects: Mechanistic complexity of the crowded milieu. Molecules.

[43] Held M, Metzner P, Prinz J-H, Noé F (2011). Mechanisms of protein–ligand association and its modulation by protein mutations. Biophys. J..

[44] Sasahara K, Uedaira H (1993). Solubility of amino acids in aqueous poly (ethylene glycol) solutions. Colloid Polym. Sci..

[45] Hirano A, Shiraki K, Arakawa T (2012). Polyethylene glycol behaves like weak organic solvent. Biopolymers.

[46] Street TO, Bolen DW, Rose GD (2006). A molecular mechanism for osmolyte-induced protein stability. Proc. Natl. Acad. Sci. USA.

[47] Gekko K (1981). Mechanism of polyol-induced protein stabilization: Solubility of amino acids and diglycine in aqueous polyol solutions. J. Biochem..

[48] Timasheff SN (2002). Protein hydration, thermodynamic binding, and preferential hydration. Biochemistry.

[49] Arakawa T, Timasheff SN (1982). Stabilization of protein structure by sugars. Biochemistry.

[50] Abriata LA, Spiga E, Peraro MD (2016). Molecular effects of concentrated solutes on protein hydration, dynamics, and electrostatics. Biophys. J..

[51] Mukherjee SK, Gautam S, Biswas S, Kundu J, Chowdhury PK (2015). Do Macromolecular crowding agents exert only an excluded volume effect? A protein solvation study. J. Phys. Chem. B.

[52] King JT, Arthur EJ, Brooks CL, Kubarych KJ (2014). Crowding induced collective hydration of biological macromolecules over extended distances. J. Am. Chem. Society.

[53] Kirby NI, DeRose EF, London RE, Mueller GA (2004). NvAssign: Protein NMR spectral assignment with NMRView. Bioinformatics.

[54] Delaglio F (1995). NMRPipe: A multidimensional spectral processing system based on UNIX pipes. J. Biomol. NMR.

[55] Ikura M, Kay LE, Bax A (1990). A novel approach for sequential assignment of proton, carbon-13, and nitrogen-15 spectra of larger proteins: heteronuclear triple-resonance three-dimensional NMR spectroscopy. Application to calmodulin. Biochemistry.

[56] Weininger U, Respondek M, Akke M (2012). Conformational exchange of aromatic side chains characterized by L-optimized TROSY-selected 13C CPMG relaxation dispersion. J. Biomol. NMR.

[57] Grzesiek S, Stahl SJ, Wingfield PT, Bax A (1996). The CD4 determinant for downregulation by HIV-1 Nef directly binds to Nef. Mapping of the Nef binding surface by NMR. Biochemistry.

[58] Gemmecker G, Jahnke W, Kessler H (1993). Measurement of fast proton exchange rates in isotopically labeled compounds. J. Am. Chem. Soc..

[59] Jones JA, Wilkins DK, Smith LJ, Dobson CM (1997). Characterisation of protein unfolding by NMR diffusion measurements. J. Biomol. NMR.

[60] Berger S, Braun S (2004). 200 and more NMR Experiments. A Practical Course.

[61] Bárcena-Uribarri I (2013). Use of nonelectrolytes reveals the channel size and oligomeric constitution of the *Borrelia burgdorferi* P66 porin. PLoS ONE.

[62] Sidebottom DL, Tran TD (2010). Universal patterns of equilibrium cluster growth in aqueous sugars observed by dynamic light scattering. Phys. Rev. E Stat. Nonlin. Soft Matter Phys..

[63] Zegarra FC (2019). Crowding-induced elongated conformation of urea-unfolded apoazurin: Investigating the role of crowder shape in silico. J. Phys. Chem. B.

[64] Eftink MR (1997). Fluorescence methods for studying equilibrium macromolecule-ligand interactions. Methods Enzymol..

[65] Lohman TM, Bujalowski W (1991). Thermodynamic methods for model-independent determination of equilibrium binding isotherms for protein–DNA interactions: Spectroscopic approaches to monitor binding. Methods Enzymol..

