# Supplementary material for: Impact of crowded environments on binding between protein and single-stranded DNA
Source: Sci Rep. 2021 Sep 3;11:17682. doi: 10.1038/s41598-021-97219-1 (PMC8417293; doi:10.1038/s41598-021-97219-1)
Supplement: Supplementary file 1 — Supplementary Figures. [file 41598_2021_97219_MOESM1_ESM.pdf]

Supplementary Information

Impact of crowded environments on binding between protein and single-stranded DNA

Birgit Köhn[1,2], Patricia Schwarz[1], Pernilla Wittung-Stafshede[3], and Michael Kovermann[1,2,*]

[1]Department of Chemistry, University of Konstanz, Universitätsstrasse 10, 78457 Konstanz, Germany

[2]Konstanz Research School Chemical Biology KoRS-CB, University of Konstanz, Universitätsstrasse 10, 78457 Konstanz, Germany

[3]Department of Biology and Biological Engineering, Chalmers University of Technology, 41296 Gothenburg, Sweden

[*]Michael Kovermann, Department of Chemistry, University of Konstanz, Universitätsstrasse. 10, 78457 Konstanz, Germany, Phone number: +49 7531 88 3801, Email: michael.kovermann@uni-konstanz.de

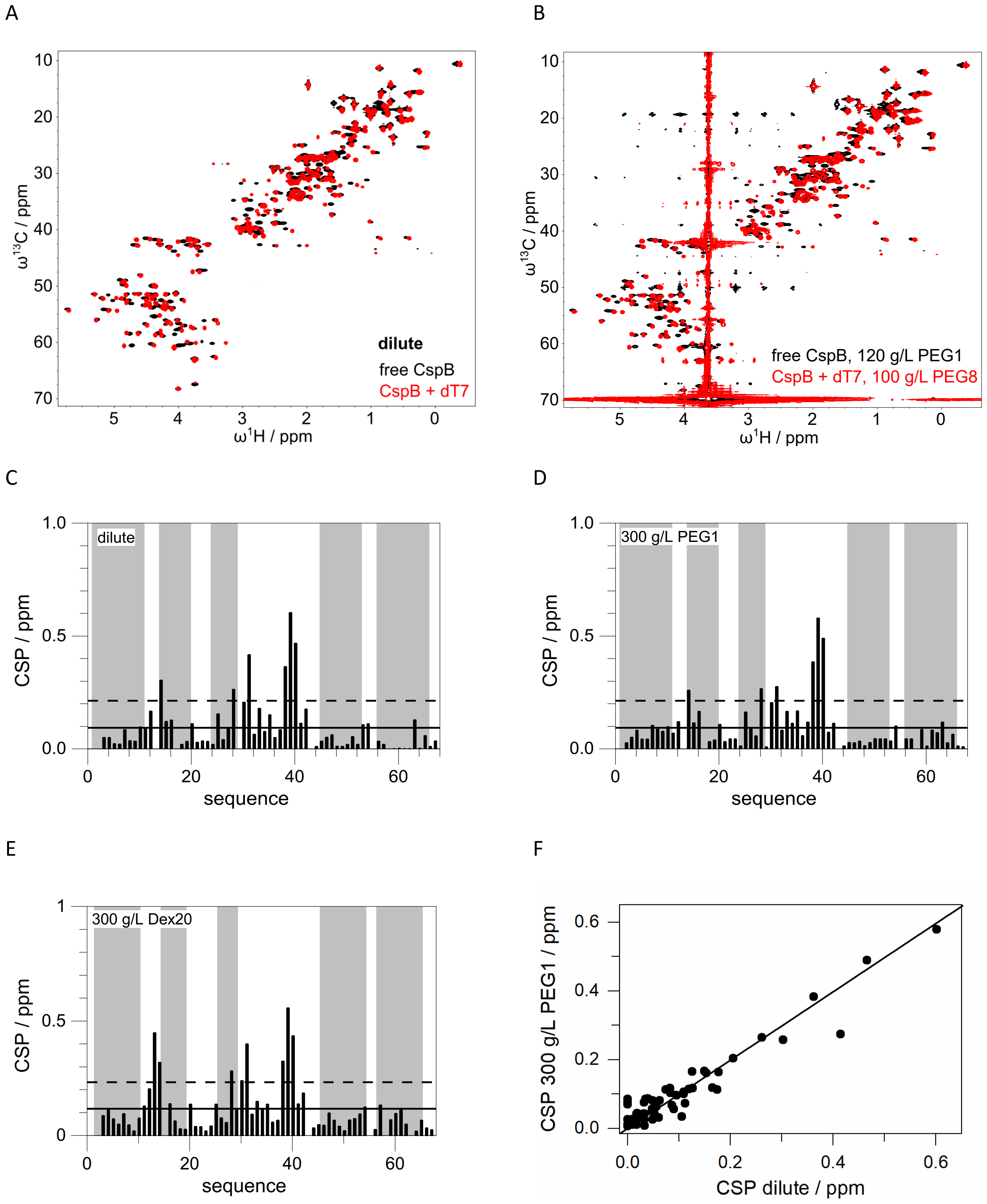
A
B
ω13C / ppm
ω1H / ppm
dilute
free CspB
CspB + dT7
free CspB, 120 g/L PEG1
CspB + dT7, 100 g/L PEG8
C
D
E
F
CSP / ppm
sequence
dilute
300 g/L PEG1
300 g/L Dex20
CSP 300 g/L PEG1 / ppm
CSP dilute / ppm

G

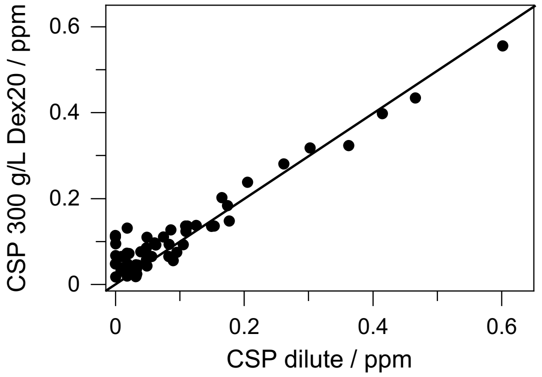
CSP 300 g/L Dex20 / ppm
0.6
0.4
0.2
0
0
0.2
0.4
0.6
CSP dilute / ppm

H

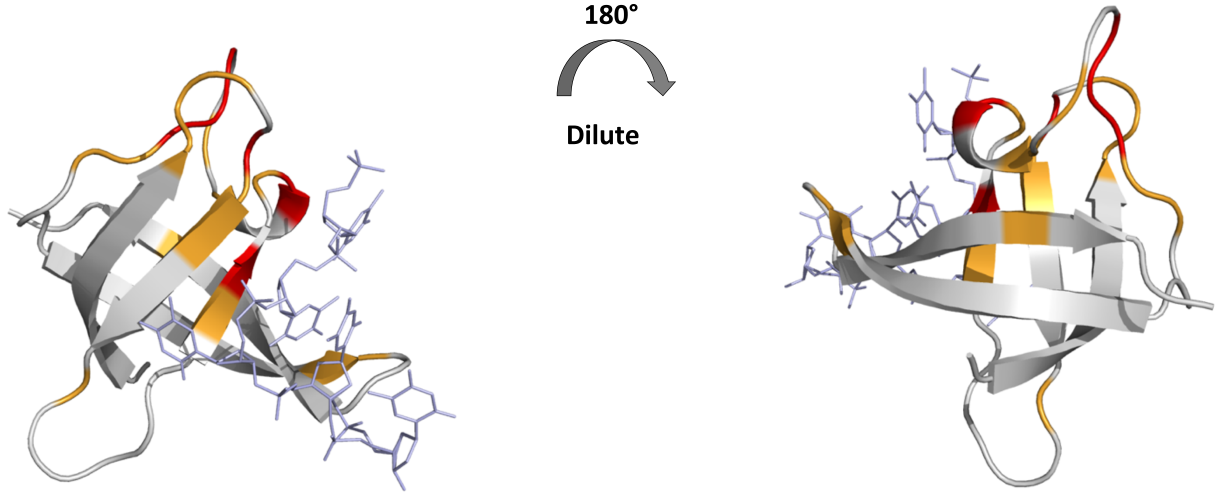
180°
Dilute

I

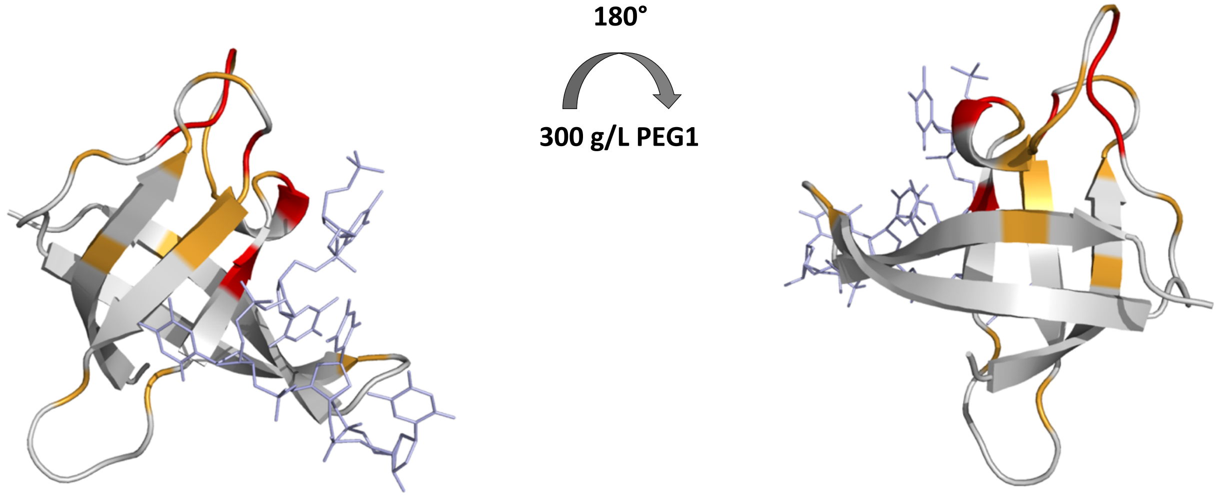
180°
300 g/L PEG1

J

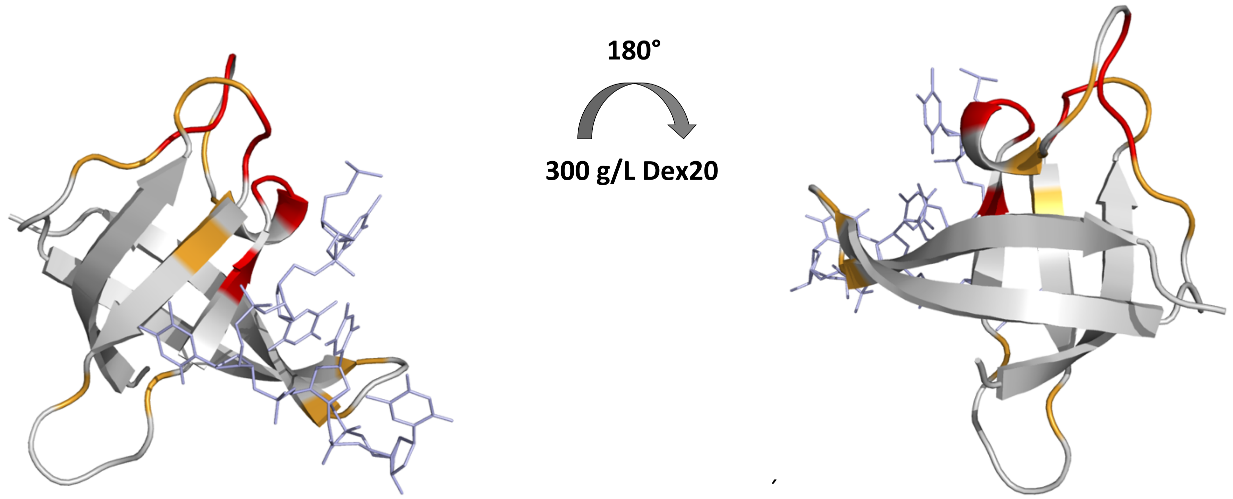

K

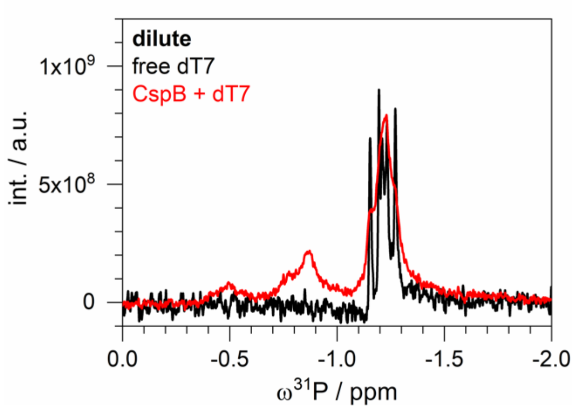

L

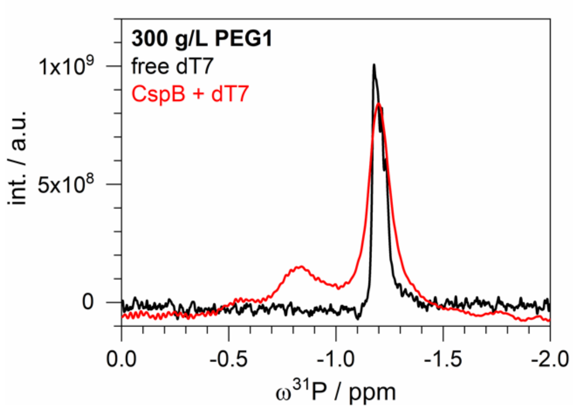

Figure S1

Probing the interaction between dT7 and *Bs*CspB in absence and presence of a crowded environment by high-resolution NMR spectroscopy. Two-dimensional heteronuclear ^{1}H-^{13}C HSQC spectra comparing free (colored in black) and dT7-bound *Bs*CspB (colored in red) under dilute conditions (A) and in presence of c = 120 g/L PEG1 for free *Bs*CspB and c = 100 g/L PEG8 representing *Bs*CspB + dT7 (B). Protein concentration was set to 477 µM in dT7-bound states. In free states, protein concentration was set to 250 µM in absence and to 800 µM in presence of 120 g/L PEG1, respectively. A twofold molar excess of dT7 regarding *Bs*CspB has been used to populate the respective ligand-bound state of *Bs*CspB. Chemical shift perturbations (CSPs) between free and dT7-bound state of *Bs*CspB have been analyzed for dilute conditions (C) and in presence of 300 g/L PEG1 (D) or 300 g/L Dex20 (E) analyzing 2D ^{1}H-^{15}N HSQC NMR spectra. The horizontal line (continuous mode) indicates the mean whereas the dotted line represents the mean plus one standard deviation. Beta sheet regions comprising *Bs*CspB are highlighted by using a background colored in gray. (F-

H) Comparison of CSP data shown in C, D, and E on a residue-by-residue basis. Structural mapping of CSP data presented in panels C-E analyzing the interaction between dT7 and *Bs*CspB in absence (H) and presence of 300 g/L PEG1 (I) or 300 g/L Dex20 (J) using pdb-file 2ES2. CSP values above the mean are coloured in orange whereas CSP values above the mean plus one standard deviation are coloured in red, respectively. The main and side chain atoms comprising the oligonucleotide dT6 present in 2ES2.pdb are colored in light blue. The structural representations have been prepared using PyMol (www.pymol.org). One-dimensional ^{31}P NMR spectra have been acquired comparing free (colored in black) and dT7-bound *Bs*CspB (colored in red) under dilute condition (K) and in presence of 300 g/L PEG1 (L). All NMR spectra have been acquired at T = 298 K and B_0 = 18.8 T.

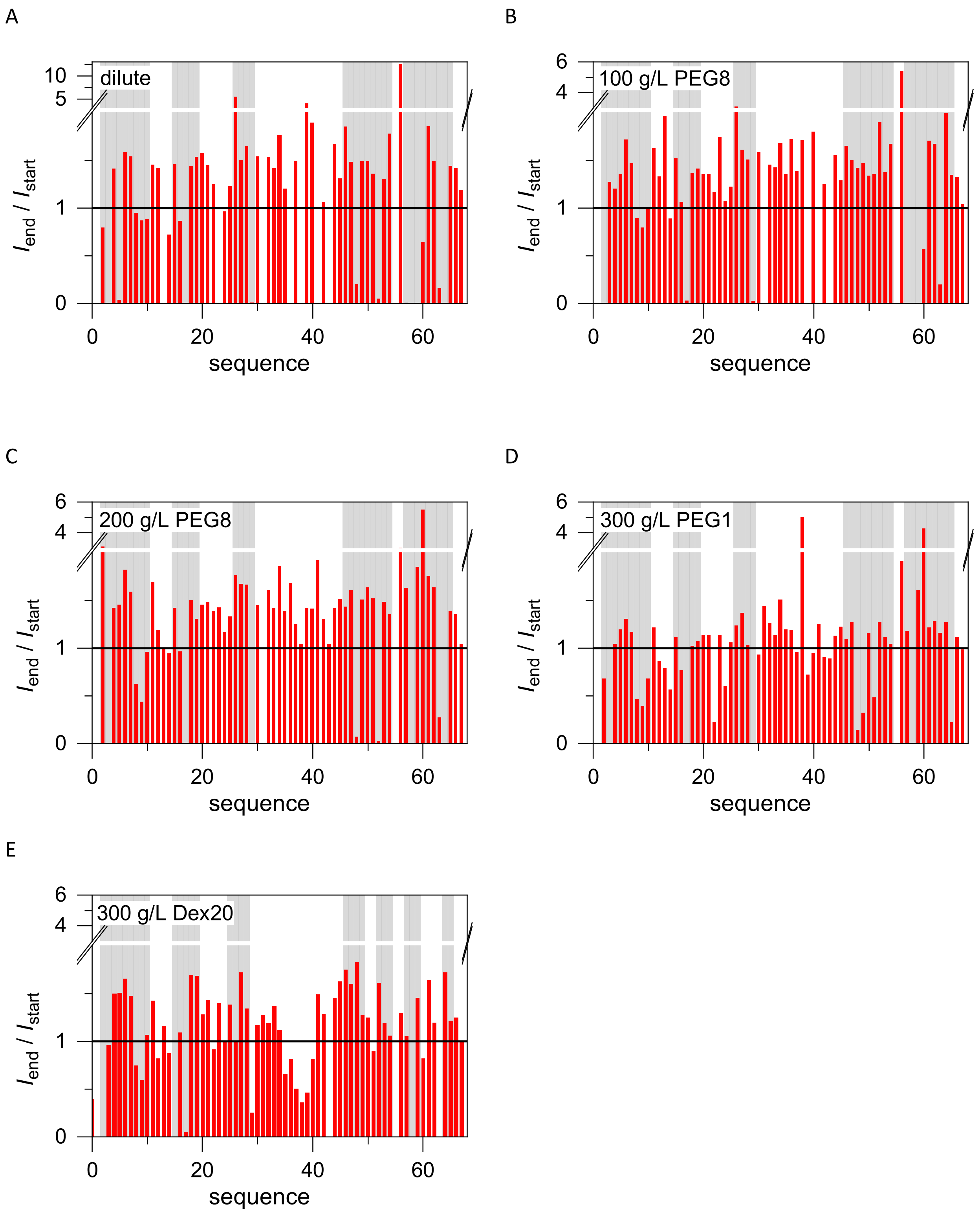

Figure S2

Ratio of intensities of signals heights of cross-peaks of *Bs*CspB present in 2D ^{1}H-^{15}N HSQC NMR spectra upon addition of a twofold excess of dT7. I_{start} denotes signal heights of all cross-peaks comprising *Bs*CspB at $c^{dT7} = 0$ µM whereas I_{end} denotes signal heights of cross-peaks at an about twofold excess of dT7

regarding to *Bs*CspB (c^{dT7} =477 µM in the absence (A) and presence of 100 g/L PEG8 (B) wheres c^{dT7} =287 µM in the presence of 200 g/L PEG8 (C), 300 g/L PEG1 (D), and 300 g/L Dex 20 (E), respectively). Beta sheet regions comprising *Bs*CspB are highlighted by using a background colored in gray according to 1NMR.pdb.

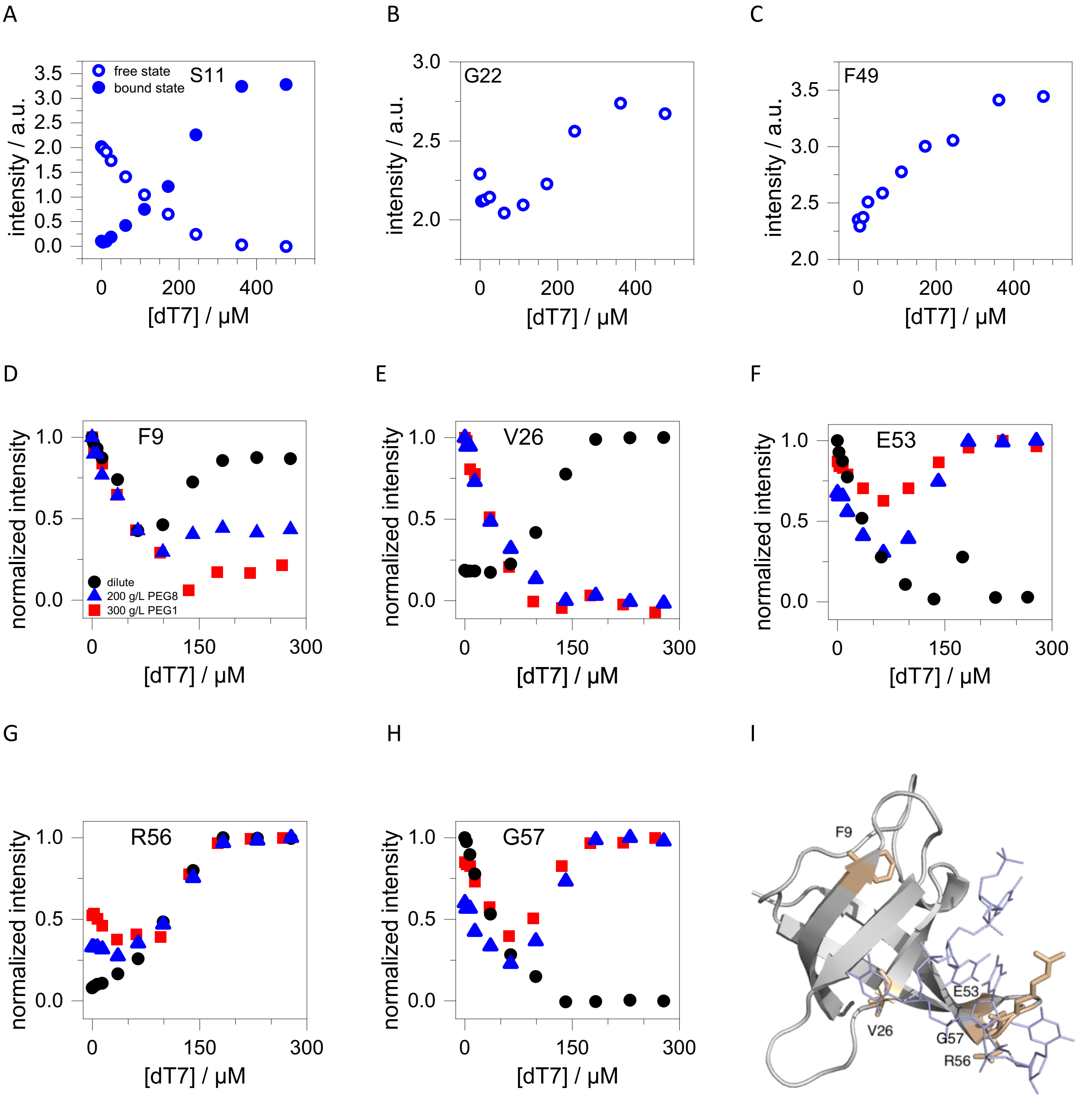

Figure S3

Analysis of the course of signal heights following the interaction of dT7 to *Bs*CspB using data obtained in two-dimensional ^{1}H-^{15}N HSQC NMR spectra comparing dilute conditions with a crowded environment. The progression of individual signal height profiles report on cross-peaks of Ser11 (A), Gly22 (B), and Phe49 (C) in presence of 100 g/L PEG8. Open circles refer to the cross-peak of Ser11 in the free state, closed circles refer to the dT7-bound state illustrating the slow exchange regime on the NMR time scale regarding this residue. Additionally, the progression of signal heights of cross-peaks of Phe9 (D), Val26 (E), Glu53 (F), Arg56 (G), and Gly57 (H) are shown applying dilute (circles, colored in black), 200 g/L PEG8 (triangles, colored in blue) and 300 g/L PEG1 condition (squares, colored in red). (I) Main and side chain atoms of F9,

V26, E53, R56, and G57 are shown in orange using 2ES2.pdb (comprising the oligonucleotide dT6 colored in light blue). The structural representation has been prepared using PyMol (www.pymol.org). All data have been acquired at T = 298 K and B_0 = 18.8 T and the titration series started at $c^{Bs\mathrm{CspB}}$ = 150 µM in all cases.

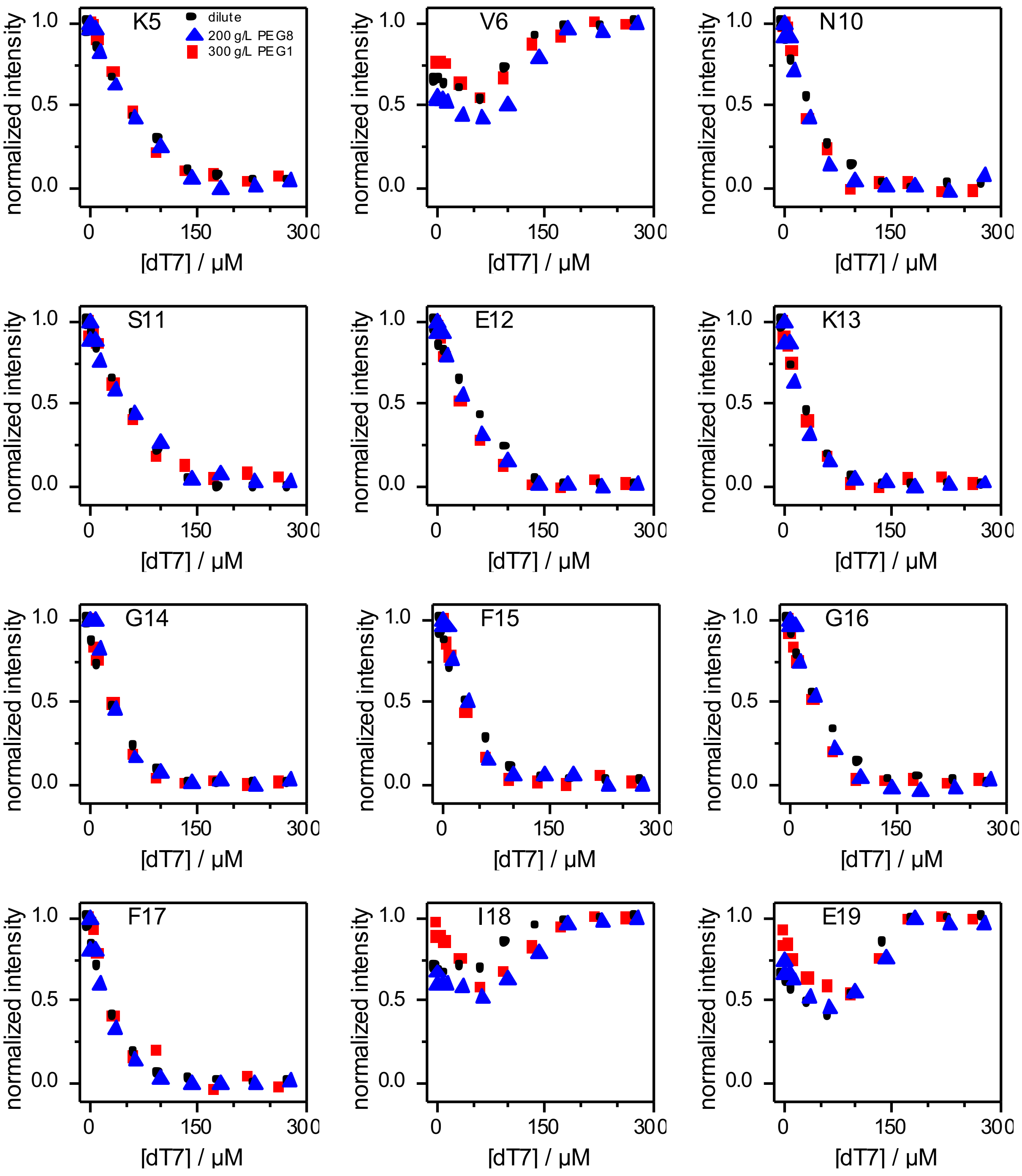
K5
dilute
200 g/L PEG8
300 g/L PEG1
V6
N10
S11
E12
K13
G14
F15
G16
F17
I18
E19
normalized intensity
[dT7] / μM

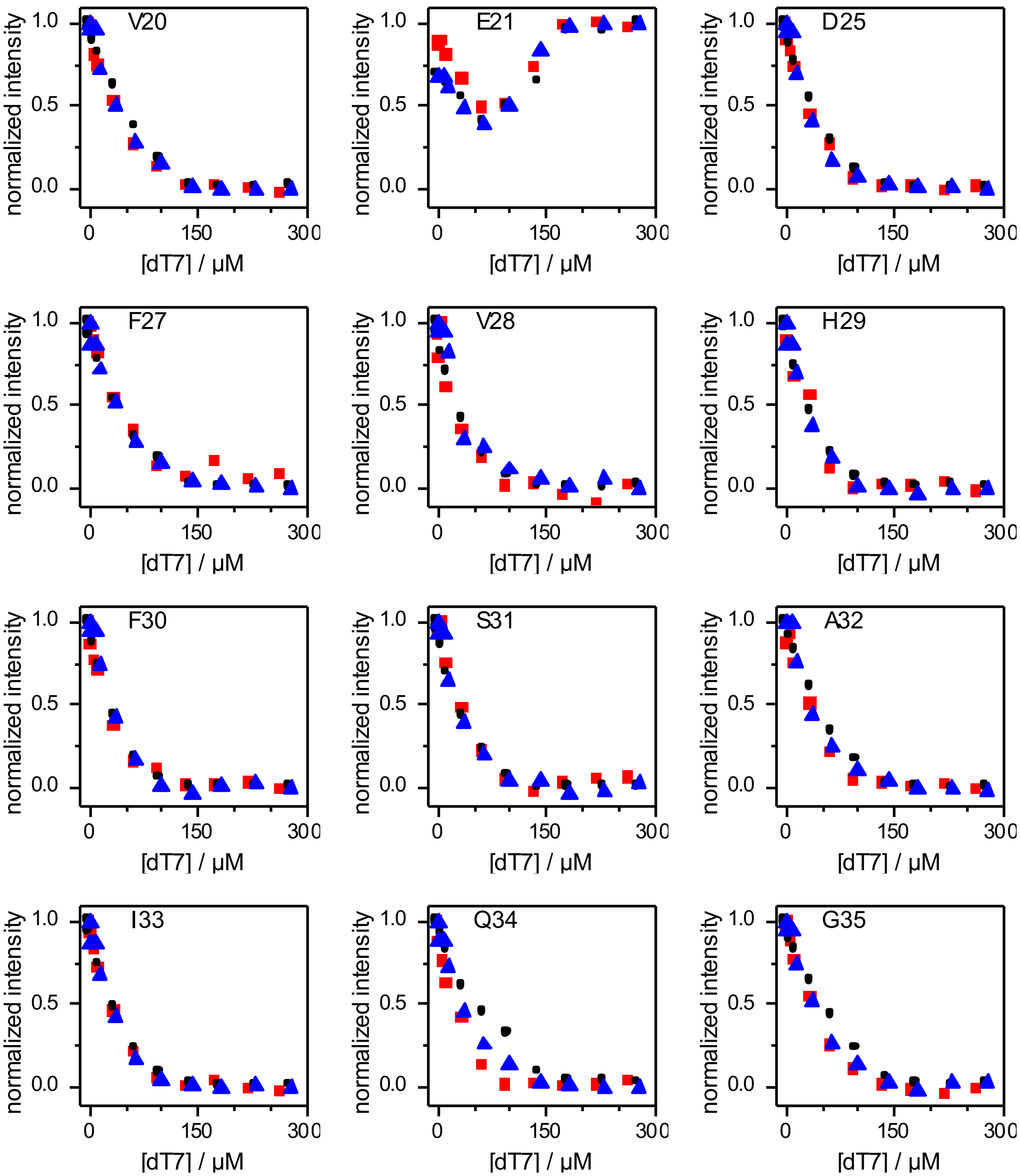
V20
E21
D25
F27
V28
H29
F30
S31
A32
I33
Q34
G35
normalized intensity
1.0
0.5
0.0
0
150
300
[dT7] / µM

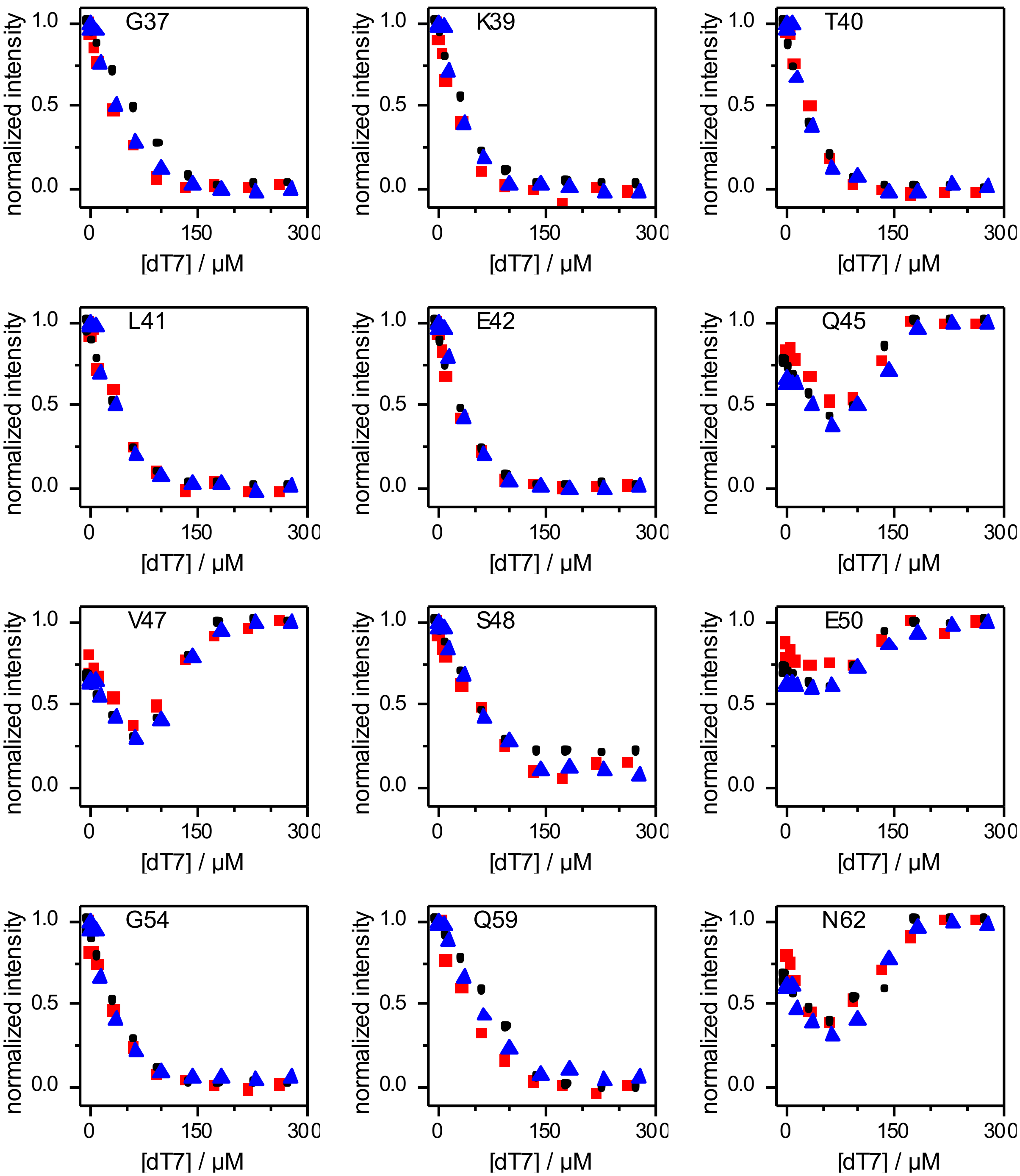
G37
K39
T40
L41
E42
Q45
V47
S48
E50
G54
Q59
N62
normalized intensity
[dT7] / μM
0.0
0.5
1.0
0
150
300

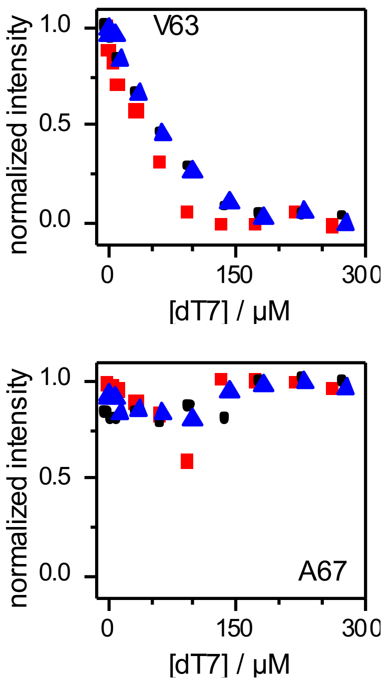

Figure S4

Analysis of the course of signal heights following the titration of dT7 to *Bs*CspB using data obtained in two-dimensional ^{1}H-^{15}N HSQC NMR spectra comparing dilute conditions (circles, colored in black) with a crowded environment (200 g/L PEG8 shown as triangles, colored in blue; 300 g/L PEG1 shown as rectangles, colored in red). All data have been acquired at T = 298 K and B_0 = 18.8 T, the titration series started at $c^{Bs\mathrm{CspB}}$ = 150 µM in all cases.

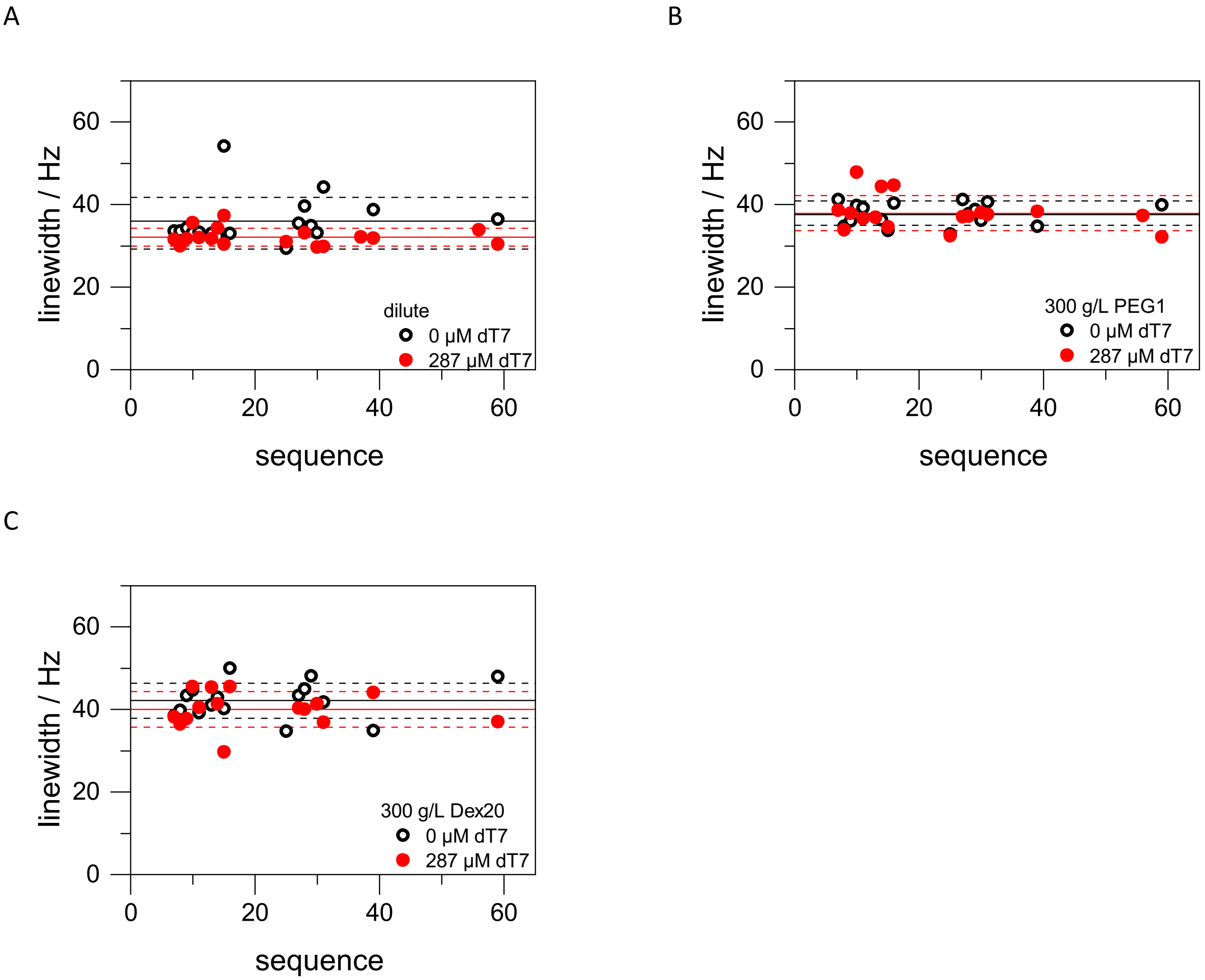

Figure S5

Analysis of line width of ^{1}H resonance signals for 17 residues (K7, W8, F9, N10, S11, K13, G14, F15, G16, D25, F27, V28, H29, F30, S31, K39, and Q59) *comprising the binding interface* between dT7 and *Bs*CspB comparing dilute (A) with crowding environments (c^{PEG1} = 300 g/L (B), c^{Dex20} = 300 g/L (C)) based on the acquisition of two-dimensional ^{1}H-^{15}N HSQC NMR data. Free state conditions are shown as open circles (colored in black) whereas crowded environments are shown as filled circles (colored in red). All NMR data have been acquired at T = 298 K and B_0 = 18.8 T. The horizontal line (continuous mode) indicates the mean whereas the dotted lines represent the mean plus or minus one standard deviation. The following numerical values have been calculated regarding the mean of line widths: 36 ± 6 Hz (free state, dilute), 32 ± 2 Hz (dT7-bound state, dilute), 38 ± 2 Hz (free state, 300 g/L PEG1), 38 ± 4 Hz (dT7-bound state, 300 g/L PEG1), 42 ± 4 Hz (free state, 300 g/L Dex20), 40 ± 4 Hz (dT7-bound state, 300 g/L Dex20).

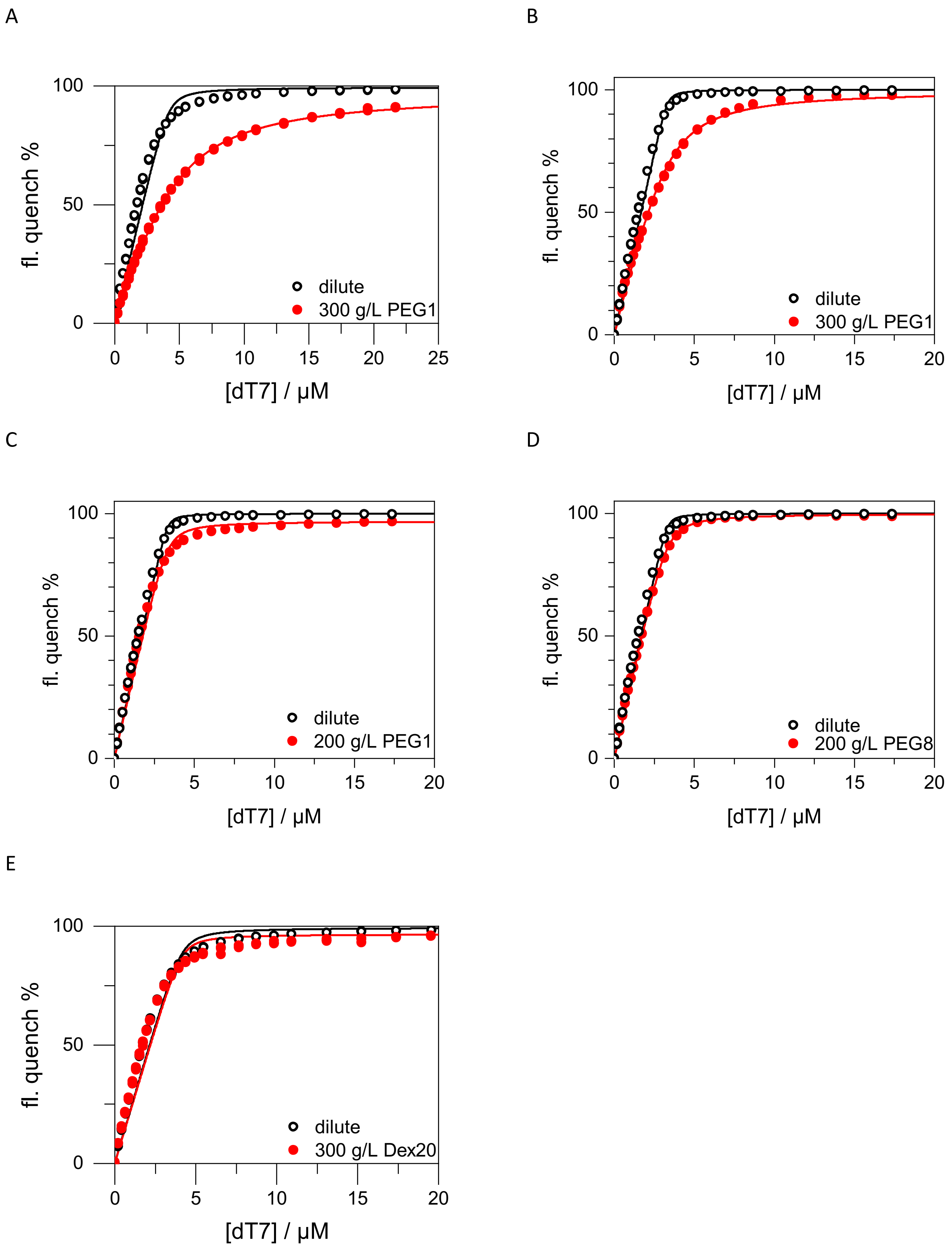
A
100
50
0
fl. quench %
0
5
10
15
20
25
[dT7] / μM
dilute
300 g/L PEG1
B
100
50
0
fl. quench %
0
5
10
15
20
[dT7] / μM
dilute
300 g/L PEG1
C
100
50
0
fl. quench %
0
5
10
15
20
[dT7] / μM
dilute
200 g/L PEG1
D
100
50
0
fl. quench %
0
5
10
15
20
[dT7] / μM
dilute
200 g/L PEG8
E
100
50
0
fl. quench %
0
5
10
15
20
[dT7] / μM
dilute
300 g/L Dex20

Figure S6

Fluorescence spectroscopy performed in equilibrium monitoring the interaction of dT7 with *Bs*CspB comparing dilute conditions (open circles, colored in black) with crowded environments (filled circles, colored in red). Equation (1) has been used to fit experimental data and is shown as continuous line. Numerical data specifying ligand binding affinity are presented in Tab. 1. Applying dilute conditions, the protein concentration was set to $c^{Bs\text{CspB}}$ = 0,5 µM in A and E, whereas $c^{Bs\text{CspB}}$ = 4 µM in B-D. Applying crowded environments, $c^{Bs\text{CspB}}$ = 0.5 µM (A), $c^{Bs\text{CspB}}$ = 4 µM (B-D), $c^{Bs\text{CspB}}$ = 2.8 µM (B-D). All fluorescence experiments have been performed at T = 298 K.

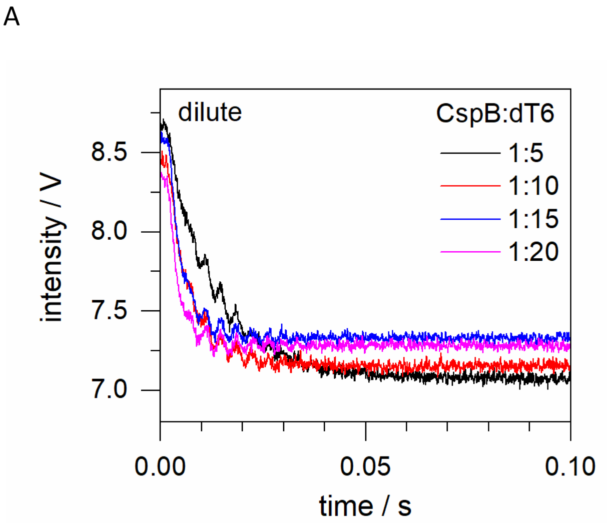

Figure S7

Raw data obtained for stopped-flow fluorescence kinetics monitoring the interaction of dT6 to *Bs*CspB on a millisecond time scale. Data have been recorded under dilute condition (A) and in presence of 300 g/L PEG1 (B) applying a constant protein concentration of $c^{Bs\text{CspB}}$ = 0.06 µM and varying ratios regarding ligand concentration color-coded in the following manner: 1:5 (colored in black), 1:10 (colored in red), 1:15 (colored in blue), and 1:20 (colored in magenta), respectively. A mono exponential function has been used to fit the intensity profiles to obtain the apparent rate constant, k_{obs}, characterizing the time-dependent change in fluorescence intensity due to the addition of dT6 to *Bs*CspB. All kinetic fluorescence experiments have been performed at T = 288 K.

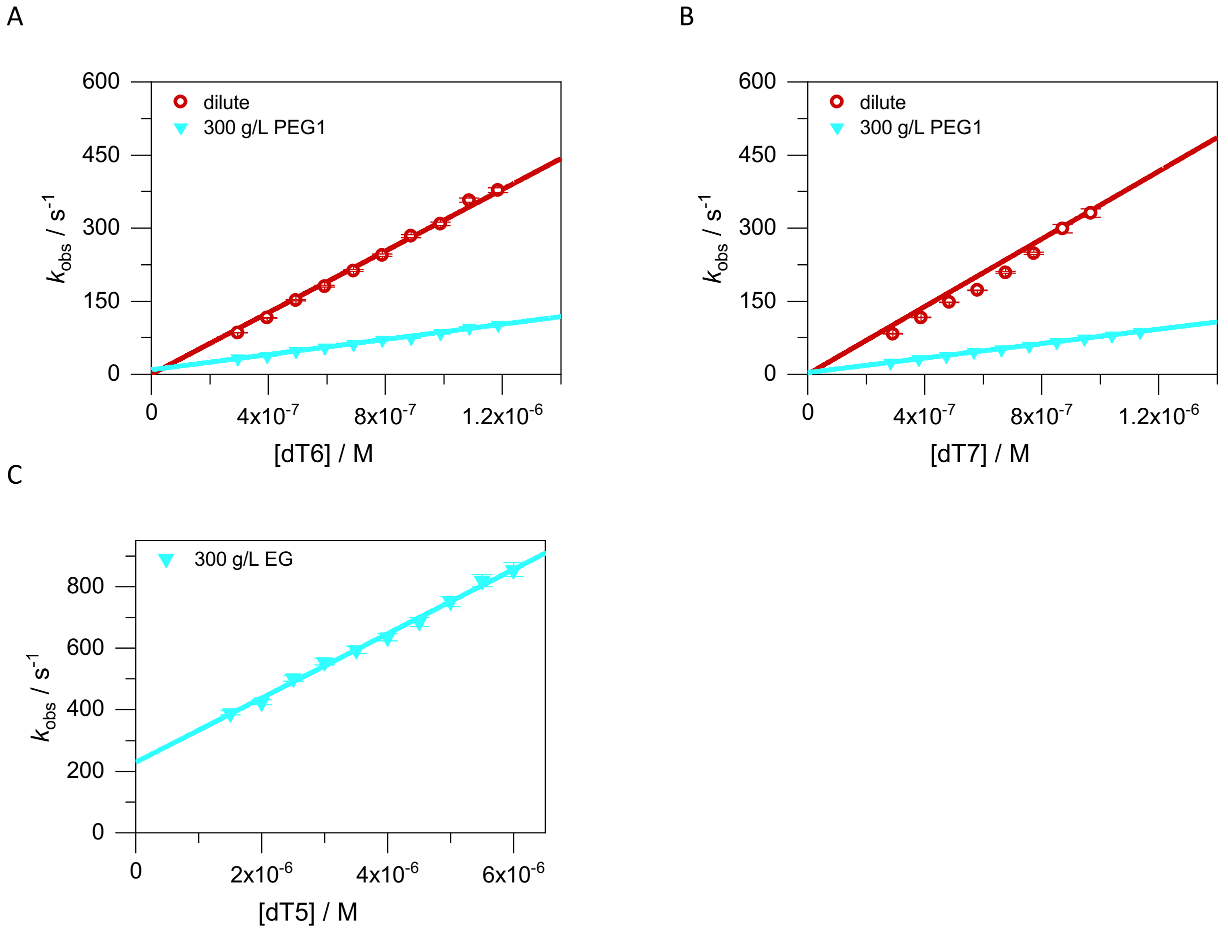

Figure S8

Applying kinetic stopped-flow fluorescence spectroscopy to probe the interaction between *Bs*CspB and dT6 (A), dT7 (B), and dT5 (C) in absence (open circles, colored in red) and in presence of crowded environments (triangles with tip to bottom, colored in cyan). All experiments have been performed at T = 288 K using $c^{Bs\text{CspB}}$ = 60 nM (A, B) except for applying c^{EG} = 300 g/L where $c^{Bs\text{CspB}}$ = 300 nM has been used (C). A linear function has been used to perform regression of experimental data regarding k_{obs} (continuous line) to obtain rate constants of ligand association, k_{on}, and dissociation, k_{off}. Numerical values obtained from the regression of experimental data are presented in Tab. 2.

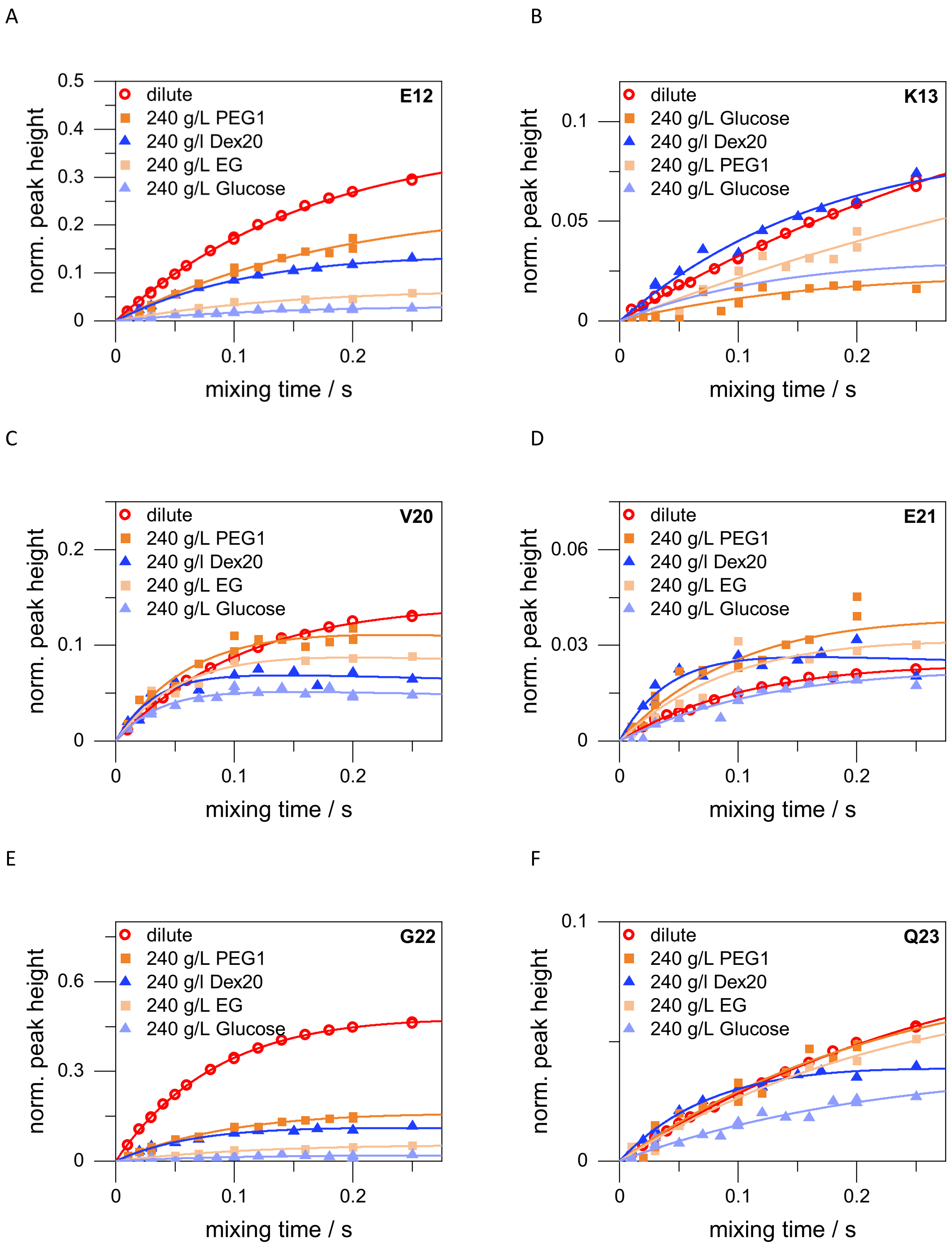
A
E12
dilute
240 g/L PEG1
240 g/l Dex20
240 g/L EG
240 g/L Glucose
norm. peak height
0.5
0.4
0.3
0.2
0.1
0
mixing time / s
0
0.1
0.2
B
K13
dilute
240 g/L Glucose
240 g/l Dex20
240 g/L PEG1
240 g/L Glucose
norm. peak height
0.1
0.05
0
mixing time / s
0
0.1
0.2
C
V20
dilute
240 g/L PEG1
240 g/l Dex20
240 g/L EG
240 g/L Glucose
norm. peak height
0.2
0.1
0
mixing time / s
0
0.1
0.2
D
E21
dilute
240 g/L PEG1
240 g/l Dex20
240 g/L EG
240 g/L Glucose
norm. peak height
0.06
0.03
0
mixing time / s
0
0.1
0.2
E
G22
dilute
240 g/L PEG1
240 g/l Dex20
240 g/L EG
240 g/L Glucose
norm. peak height
0.6
0.3
0
mixing time / s
0
0.1
0.2
F
Q23
dilute
240 g/L PEG1
240 g/l Dex20
240 g/L EG
240 g/L Glucose
norm. peak height
0.1
0
mixing time / s
0
0.1
0.2

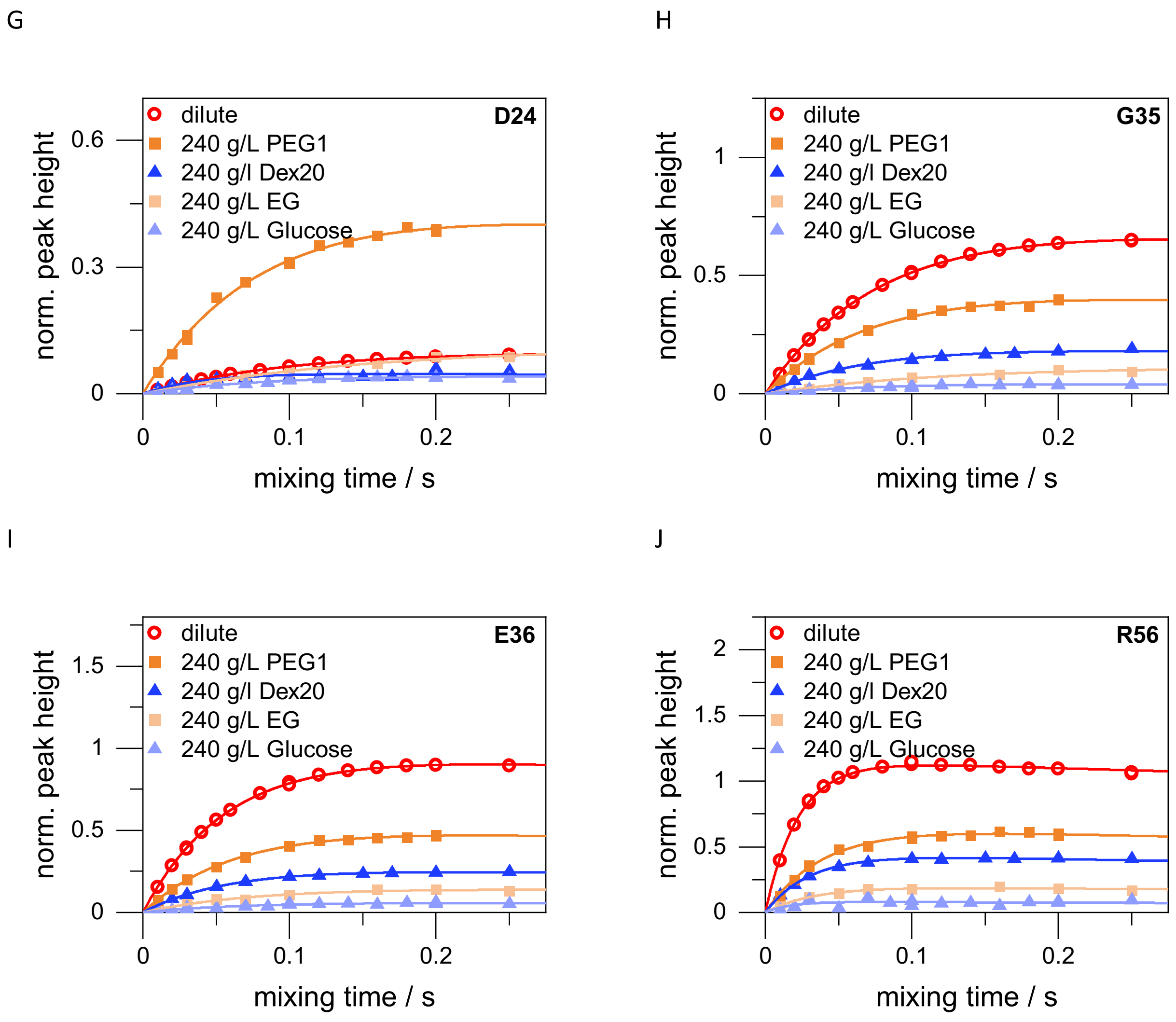

Figure S9

Build-up of signal heights of cross-peaks comprising *Bs*CspB present in 2D ^{1}H-^{15}N HSQC spectra applying a modified MEXICO pulse sequence under dilute conditions (circles, colored in red) and different crowding environments: 240 g/L PEG1 (rectangles, colored in orange), 240 g/L EG (rectangles, colored in light orange), 240 g/L Dex20 (triangles, colored in blue), and 240 g/L glucose (triangles, colored in light blue). Panels A-J show highly affected residues comparing dilute conditions with crowded environments analyzing the rate constant of exchange, k_{ex}, between amide and solvent protons highlighted in Fig. 4A. Numerical values reporting on k_{ex} values are shown in Fig. 4B, C.

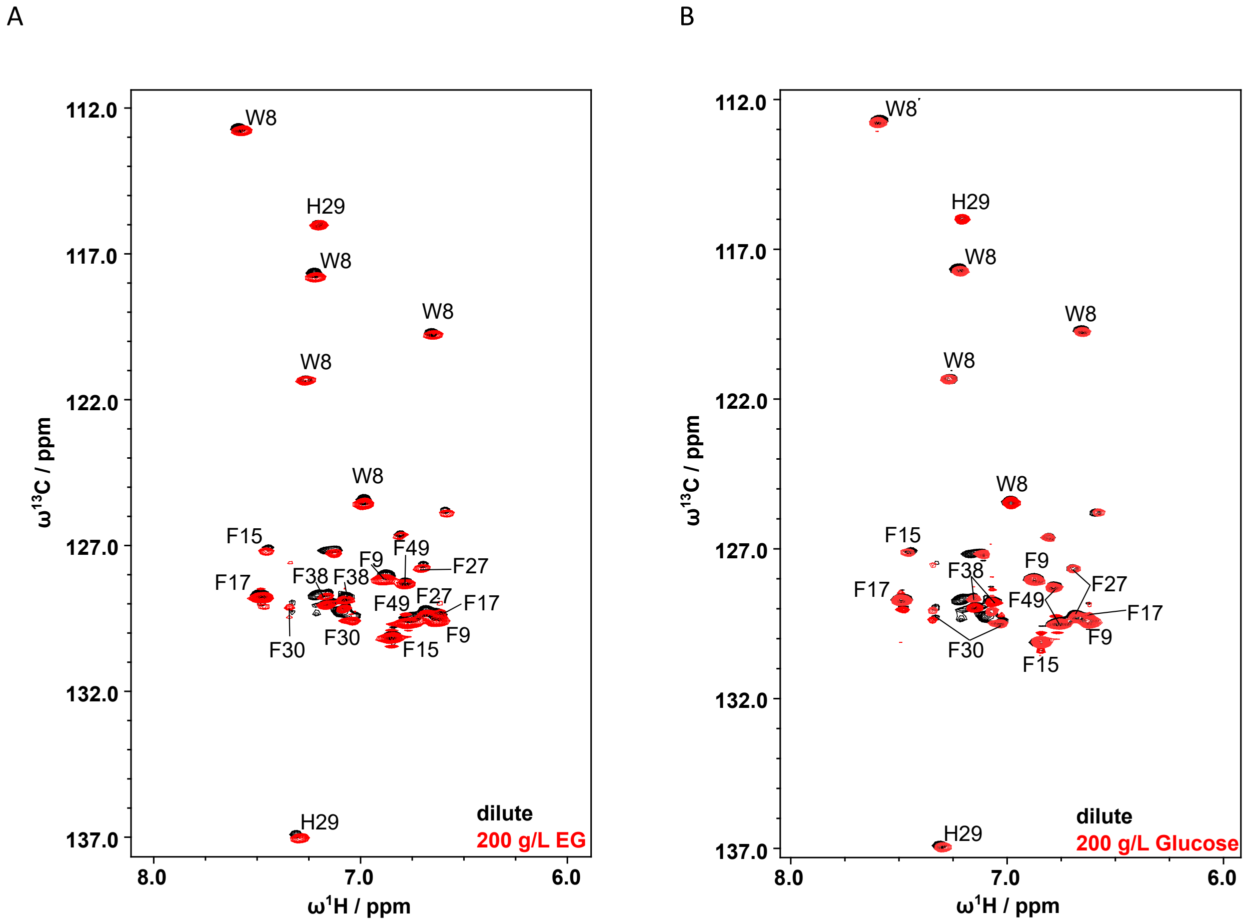

Figure S10

Two-dimensional heteronuclear ^{1}H-^{13}C HSQC NMR spectra of cross-peaks reporting on aromatic residues comprising *Bs*CspB acquired under dilute and crowded conditions. (A) Comparing dilute (colored in black) with c = 200 g/L EG (colored in red). (B) Comparing dilute (colored in black) with c = 200 g/L glucose (colored in red). The assignment of cross-peaks of *Bs*CspB is indicated by using the one letter code for amino acids followed by the position in the primary sequence. The NMR spectra have been acquired at T = 298 K and B_0 = 14.1 T.
